# A molecular scaffold for concurrent targeting of plasma and mitochondrial membranes

**DOI:** 10.1039/d5sc06276d

**Published:** 2026-01-16

**Authors:** Youbo Lai, Yi Yang, Yuping Zhao, Tony D. James, Weiying Lin

**Affiliations:** a Institute of Optical Materials and Chemical Biology, Guangxi Key Laboratory of Electrochemical Energy Materials, School of Chemistry and Chemical Engineering, Guangxi University Nanning 530004 Guangxi P. R. China weiyinglin2013@163.com; b Department of Chemistry, University of Bath Bath BA2 7AY UK t.d.james@bath.ac.uk; c School of Chemistry and Chemical Engineering, Henan Normal University Xinxiang 453007 P. R. China

## Abstract

The ability to specifically sense and image plasma membranes and mitochondrial membranes with fluorescent probes is paramount for the visualization and mechanistic understanding of these fundamental, dynamic cellular compartments. However, dual-targeting membrane probes combining a fluorophore and two distinct targeting ligands face synthetic challenges and potential functional group interference. Therefore, we designed a molecular scaffold (dual-targeting ligand) that combines both a mitochondrial anchor and a plasma membrane protein ligand to simultaneously localize at both membranes. Using this molecular scaffold, we engineered a series of fluorescent probes, T-1 to T-5. Furthermore, we demonstrate the functional applications of two probes from this series, T-1 and T-4. Owing to the distinct physicochemical properties of the targeted plasma and mitochondria membrane, T-1 can differentiate multiple cell states, including live, dead, and early apoptotic cells. Additionally, T-4, designed as a dual-targeting photosensitizer, induces cancer cell necrosis by simultaneously rupturing the plasma membrane and inducing mitochondrial swelling, leading to enhanced photosensitizing efficiency. Significantly, this research advances the development of fluorescent probe based labeling strategies and provides effective tools for biochemical and biomedical applications.

## Abstract 2

The ability to specifically sense and image plasma membranes and mitochondrial membranes with fluorescent probes is paramount for the visualization and mechanistic understanding of these fundamental, dynamic cellular compartments.

## Graphical abstract

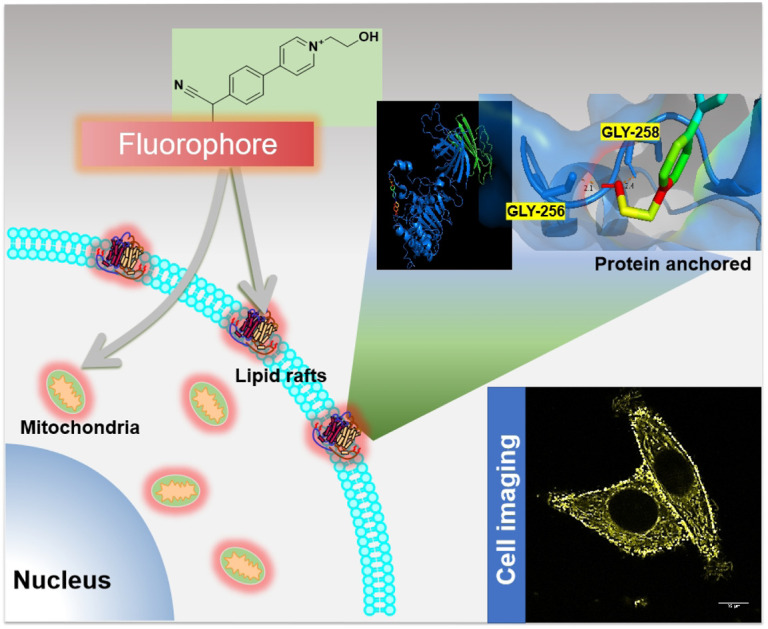

## Introduction

Biological membranes are ubiquitous, complex lipid structures that include plasma membranes and organelle membranes, serving as platforms for the exchange of diverse biological signaling molecules.^1–3^ The targeted staining of biomembranes using fluorescent probes facilitates the visualization of specific plasma membranes and organelle membranes.^4–6^ Such biomembrane specific fluorescent probes enable researchers to specifically monitor biomembranes of interest.^7–9^ As such, visualization techniques *via* optical imaging using membrane anchoring fluorescent probes that are logically designed can provide information on the location and morphology of plasma and organelle membranes, enabling the monitoring of important transient chemical messengers, biological states and dysfunction.^10–12^ Therefore, to extend the availability of specific membrane-targeting probes, various methods have been used to develop specific membrane-targeting ligands.^13^ For example, morpholine is widely used as a lysosome-targeting group;^14^ a methyl sulfonamide moiety is often selected as the ER-targeting group;^15–20^ and a phenylsulfonamide unit is utilized as a Golgi targeting moiety.^21–23^

**Fig. 1:**
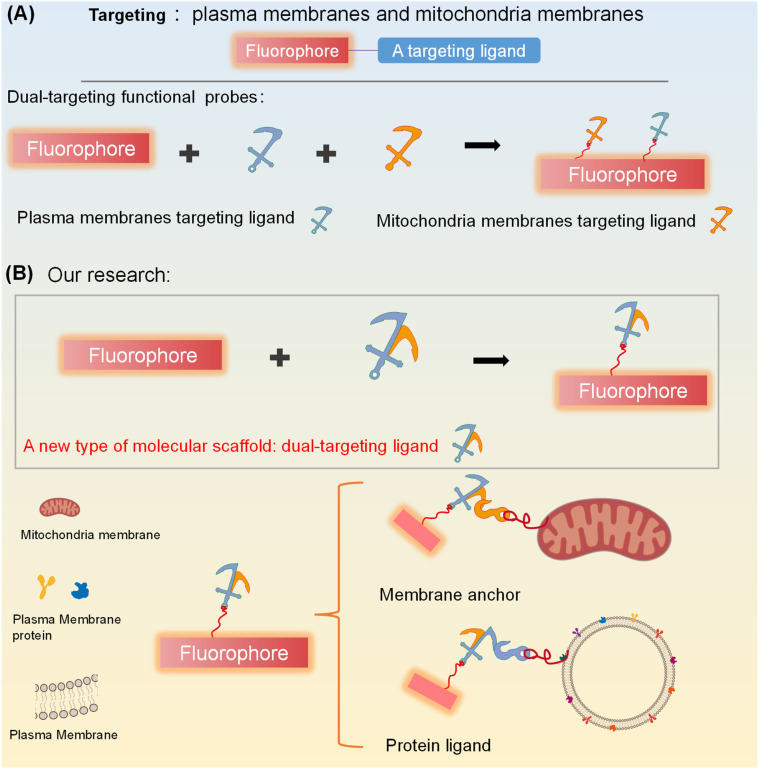
Illustration of the design strategy for membrane-targeting fluorescent probes: (A) generic scheme for dual-targeting membrane probes combining a fluorophore with two distinct membrane-targeting ligands for targeting plasma membranes and mitochondria membranes; (B) our approach for dual-targeting fluorescent probes, combining a fluorophore with a new type of dual-targeting ligand for targeting the plasma membrane and mitochondrial membranes.

In recent years, simultaneous dual targeting has aroused widespread interest among researchers.^24–28^ Therefore, simultaneous and discriminative visualization of two different membrane structures using dual targeting membrane fluorescent probes has attracted significant attention. Generic dual targeting fluorescent probes are composed of a fluorophore core and two distinct membrane-targeting molecular ligands, enabling simultaneous targeting of both the plasma membrane and the mitochondrial membrane (Fig. 1A).^29^ However, the conjugation of two distinct membrane-targeting ligands to a single fluorophore often gives rise to inherent limitations, including signal cross-talk, increased synthetic complexity, significant steric hindrance, and undesirable perturbations to the fluorophore's optical properties—collectively complicating the probe design and performance. To address these challenges and streamline the structure–function relationship between the fluorophore and targeting moieties, the development of a single molecular ligand with dual membrane-targeting capabilities emerges as a pivotal strategy. This design not only eliminates the aforementioned interferences arising from multiple discrete ligands but also simplifies synthesis, minimizes steric constraints, and preserves the fluorophore's intrinsic optical performance—ultimately enabling more precise, efficient, and reliable membrane-targeted imaging.

Currently no design strategy exists, for the construction of simultaneous dual-targeting fluorescent probes using a single molecular scaffold. Herein, we propose a rational strategy for developing a molecular scaffold for targeting mitochondria and plasma membranes (Fig. 1B). Based on this strategy, we obtained a dual-targeting ligand for targeting the mitochondria and plasma membranes, which was then used to engineer two dual targeting fluorescent probes T-1 and T-4. Compared with a single-targeting probe, T-1 can distinguish multiple cell states including live cells, dead cells, and early apoptotic cells *via* a distinct fluorescence response to different biomembranes. Notably, T-4, as a simultaneous dual targeting photosensitizer, can enhance the efficiency of the photosensitizer and may offer a new design strategy for photosensitizers.

## Results and discussion

### Strategy for developing a dual targeting group and theoretical validation

**Fig. 2:**
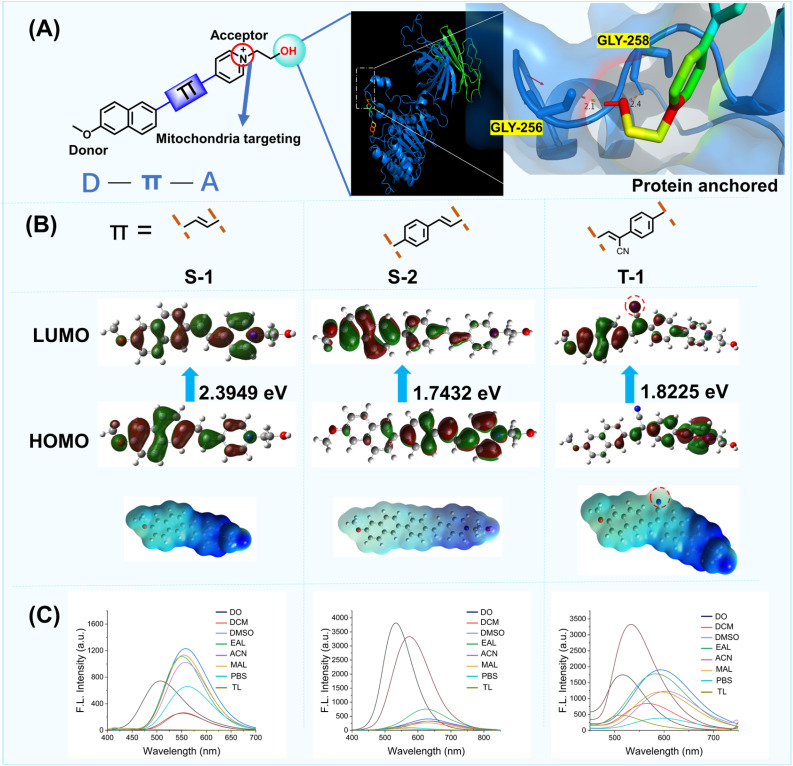
Design strategy of a single molecular group for the simultaneous targeting of two different organelles. (A) Schematic representation of specific simultaneous dual targeting group. Molecular docking calculations of the dual targeting group to GPI-anchored protein. GPI-anchored protein link integrated membrane proteins to cytoskeletal protein networks and play key roles in a variety of cellular processes. (B) Molecular structures, density functional theory calculations with LUMOs and HOMOs of S-1, S-2 and T-1. Cyanide location shown by a red dashed circle. (C) Emission spectra of S-1, S-2 and T-1 in different solvents. 1,4-Dioxane (DO), Dichloromethane (DCM), Dimethyl Sulfoxide (DMSO), Ethyl Alcohol (EAL), Acetone (ACN), Methanol (MAL), phosphate buffered saline (PBS), Tetrahydrofuran (THF), Toluene (TL).

To achieve selective targeting for two distinct lipid membranes with a single molecular scaffold, we used a rational strategy based on three features, the dual targeting group must: 1. contain two specific membrane-targeting sites, 2. be easily modifiable to permit incorporation of different functions and properties. 3. exhibit good biocompatibility and can enter cells rapidly. Based on these characteristics, we designed a single molecular scaffold for targeting mitochondria and plasma membranes. Firstly, considering that the mitochondrial membrane has negative membrane potential,^19^ we selected a molecular group with a positive charge to target the mitochondria. Therefore, a positively charged pyridinium group was incorporated into the probe (Fig. 2A). Secondly, the ligands of protein glycosylphosphatidylinositol (GPI) of plasma membranes contain many hydroxyl groups. Therefore, we envisioned that the single group should contain a hydroxyl to bind with the GPI-anchored protein for targeting plasma membranes. Considering these important design criteria, we decided to link the positively charged pyridinium with a hydroxyl to form a dual targeting unit, with the aim of achieving the simultaneous targeting of two different lipid membranes, the mitochondria and plasma membranes. Subsequently, we combined the electron donor 2-methoxynaphthalene with the dual targeting group (1-(2-hydroxyethyl)pyridin-1-ium) using π spacers. Furthermore, to investigate the binding affinity between the dual targeting group and GPI-anchored protein (PDB entry: 6ob0), we determined that the Gibbs binding energy for the system was 9.8 kJ mol^−1^ using molecular docking calculations (AutoDock 4.2). The theoretical calculations revealed that the hydroxyl group was inserted into a deep pocket delineated by the main chains of GLY-258, which confirmed that the dual targeting group exhibited excellent binding for the GPI-anchored protein through two hydrogen bonds. Using our strategy, a dual targeting group for the plasma and mitochondrial membranes was obtained, therefore, enabling the construction of dual targeted fluorescent probes S-1, S-2, and T-1 (Fig. 2B).

All compounds were synthesized as outlined in Scheme S1 and S2 (SI). First, an aldehyde group was introduced into the aromatic precursor *via* the Vilsmeier–Haack reaction (using POCl_3_/DMF as the reagent system). Subsequently, using the cyano-containing pyridine derivative P-1 as the probe moiety, a conjugated intermediate was constructed through a Knoevenagel-type condensation reaction. Finally, the target product was obtained as the nitrogen atom in the pyridine molecule attacks the carbon atom of 2-iodoethanol *via* its lone-pair electrons, which forms a new chemical bond and displaces iodide ions (I^−^). The synthetic process relies on typical organic reactions including condensation, nucleophilic addition, and formylation; structural diversification of T-1 to T-5 was achieved by employing aldehyde precursors with distinct frameworks. The proposed structures were readily characterized by analytical data including ^1^H NMR, ^13^C NMR, and high-resolution mass spectrometry (Fig. S23–S44, SI).

As shown in Fig. 2B, probe S-1 incorporates an alkene as the π-bridge. Geometry optimization and computation of the Highest Occupied Molecular Orbital (HOMO)– Lowest Unoccupied Molecular Orbital (LUMO) gap (2.3949 eV in ethanol) were performed using DFT/B3LYP/6-31G(d,p) (Gaussian 09W). The resulting electrostatic potential diagram revealed the molecular orbital arrangement and confirmed the intramolecular charge transfer (ICT) nature of S-1. We then determined the fluorescence spectra of S-1 in various solvents. The fluorescence wavelength of S-1 was red shifted with an increase in the solvent polarity. To enhance the lipophilicity and extend the emission wavelength of S-1, we inserted a benzene group at the π spacer to generate S-2. As expected, using the benzene π spacer, the HOMO–LUMO energy gap of S-2 is reduced to 1.7432 eV and the emission wavelength increased from 550 to 620 nm in ethanol (Fig. 2C). However, due to delocalized π-bonds of the benzene ring which are higher in energy and exhibit low polarizability, S-2 exhibits poor optical properties. Therefore, to improve the system, we introduced a cyanide group to the π spacer of S-2 to generate T-1 (Fig. S3). To validate our assumption, T-1 was evaluated using Gaussian 09W software. Interestingly, owing to the polar cyano group in the π spacer, the HOMO and LUMO overlap was reduced resulting in a larger Stokes shift (223 nm) compared to the Stokes shift (198 nm) of S-2 in phosphate buffered saline (PBS). These results indicated that the cyanide group of T-1 increased the electron withdrawing nature and electronegativity. Additionally, the cyanide group increases the number of hydrogen bonding sites in the molecule and increases the hydrophilicity and improves the solubility.

### Evaluation of subcellular localization and verification of the simultaneous dual targeting design strategy

To verify the dual targeting design, co-localization imaging of S-1, S-2 and T-1 were performed in cells. Before imaging in cells, we evaluated the cytotoxicity of S-1, S-2, and T-1 toward the cells using a CCK8 kit (Fig. S4–6). The cell viability after 24 h incubation with 20 µM of the probes were above 85%, indicating that S-1, S-2, and T-1 exhibit low cytotoxicity and are suitable for cell imaging. In colocalization experiments using the commercial dyes DiI (yellow channel) and MitoTracker™ Deep Red FM (red channel), S-1 exhibited low colocalization between the plasma membrane and mitochondrial signals, with Pearson's correlation coefficients (PCC) of 0.046 and 0.589, respectively (Fig. S7A). S-2 similarly showed low colocalization, with PCC values of 0.046 for plasma membranes and 0.461 for the mitochondria (Fig. S7B).

**Fig. 3:**
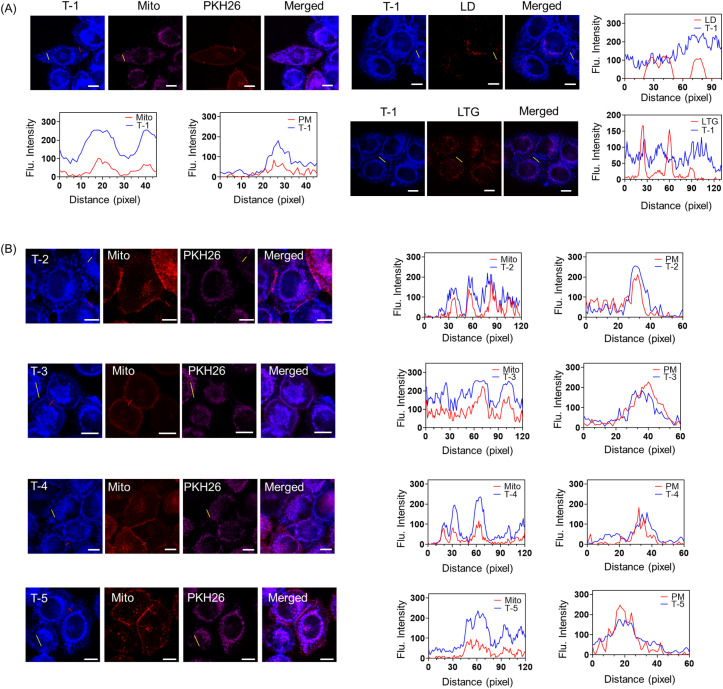
Mitochondria and plasma membrane targeting in cells. (A) Colocalization of T-1-stained compartments in HeLa cells with commercial dyes (Mito, 500 nM, *λ*_ex_ = 633 nm/*λ*_em_ = 640–660 nm; PKH26, 1 µM, *λ*_ex_ = 550 nm/*λ*_em_ = 560–600 nm; LTG, 100 nM, *λ*_ex_ = 504 nm/*λ*_em_ = 510–560 nm and BODIPY 493, 500 nM, *λ*_ex_ = 493 nm/*λ*_em_ = 503–553 nm). The inset diagrams in (A) represent the linear analysis of the selected regions of the HeLa cells after staining with T-1 and different probes. Scale bar: 10 µm. (B) Colocalization images of HeLa cells stained with 1 µM T-1 derivatives T-2 (*λ*_ex_ = 405 nm, *λ*_ex_ = 600–650 nm); T-3 (*λ*_ex_ = 405 nm, *λ*_ex_ = 600–650 nm); T-4 (*λ*_ex_ = 460 nm, *λ*_ex_ = 600–650 nm); T-5 (*λ*_ex_ = 460 nm, *λ*_ex_ = 650–700 nm) and different commercial probes including Mito (500 nM, *λ*_ex_ = 633 nm/*λ*_em_ = 640–660 nm), PKH26 (1 µM, *λ*_ex_ = 550 nm/*λ*_em_ = 560–600 nm), LTG (100 nM, *λ*_ex_ = 504 nm/*λ*_em_ = 510–560 nm), and BODIPY 493 (500 nM, *λ*_ex_ = 493 nm/*λ*_em_ = 503–553 nm). The inset diagrams in (B) represent the linear analysis of the selected regions of the HeLa cells after staining with T-1 derivatives(T-2 to T-5) and different probes. Scale bar: 10 µm.

We then performed colocalization experiments to further verify the subcellular localization of probe T-1. Colocalization experiments between T-1 and commercial probes including Mito-Tracker Deep Red (Mito, mitochondrial probe), PKH26 (plasma membrane probe), Lyso-Tracker Green (LTG, lysosomal probe) and BODIPY 493 (LD, lipid droplet probe) revealed that the blue channel fluorescence of T-1 overlapped with the pink fluorescence of Mito and the red channel fluorescence of PKH26 (Fig. 3A and S8 and 9). By contrast, the blue channel fluorescence of T-1 is not overlapped with the red channel fluorescence of LD and LTG, demonstrating its specific plasma membrane and mitochondria dual-targeted capability). Probe T-1 enabled simultaneously three-dimensional (3D) imaging of the plasma membranes and mitochondria, to provide an opportunity to simultaneously visualize the mitochondria and plasma membranes from a spatiotemporal perspective (Fig. S9). From these results, the cyanide group is an essential part of the simultaneous dual targeting probe. Furthermore, we found that using (4-(4-(cyanomethyl)phenyl)-1-(2-hydroxyethyl) pyridin-1-ium) enables attachment to plasma membranes and mitochondrial membranes with excellent membrane-permeability. These results validate our strategy for designing a new dual targeting ligand (4-(4-(cyanomethyl)phenyl)-1-(2-hydroxyethyl)pyridin-1-ium). Due to the rigid structure of naphthalene in T-1, the atoms of T-1 are located on the same plane. As such, the coplanar structure of T-1 can easily insert into the deep pockets of GPI-anchored protein. To demonstrate the influence of the steric effects on the dual targeting group, we synthesized T-2 by changing the naphthalene structure to dihydronaphthalene. Due to the single bonds of dihydronaphthalene, T-2 exhibits a more curved molecular structure than T-1. We initially measured the absorption and emission spectra of T-2 in various solvents (Fig. S10). Fig. 3B indicates that the blue channel fluorescence of T-2 overlapped with the pink fluorescence of Mito and the red channel fluorescence of PKH26, revealing that the steric effects have no effect on the simultaneous dual targeting strategy. Since halogens may exhibit considerable influence on the properties of molecules, we added halogens to the probe to generate T-3. The absorption and emission spectra of T-3 were evaluated (Fig. S11) and then T-3 was used to image cells, which indicated that the halogenated molecule T-3 exhibited simultaneous dual targeting features (Fig. 3B).

To demonstrate the versatility of the dual targeting group, we further constructed two novel dual targeting fluorescent probes T-4 and T-5 (Fig. S12 and S13). As expected, Fig. 3B demonstrates that the blue-channel fluorescence of T-4 and T-5 overlaps with both the pink fluorescence of MitoTracker (Mito) and the red-channel fluorescence of PKH26, revealing that T-4 and T-5 enable dual-targeted imaging of live cells. These observations confirm that our simultaneous dual targeting design strategy was feasible.

### Monitoring of cell states using the simultaneous dual targeting fluorescent probes

Imaging agents exhibit great potential for monitoring chemotherapeutic responses, early diseases diagnosis and disease surveillance, and can be used to directly visualize the cell states in cells. Accurate identification of multiple cell states is required for cell viability monitoring. The monitoring of cell viability has been achieved using single-targeting probes to illustrate two cell states using targeted migration to plasma membranes and organelle membranes.^30–32^ However, it is challenging to develop fluorescent probes that can stain plasma membranes and organelle membranes in multiple cell states.

**Fig. 4:**
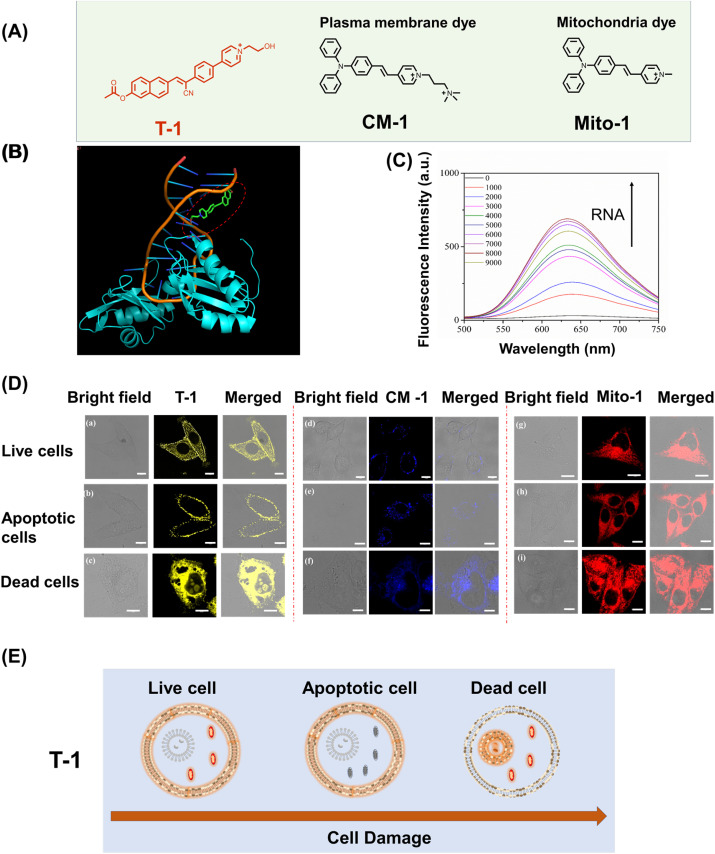
Imaging of multiple cell states using a simultaneous dual targeting fluorescent probe and single-targeting fluorescent probes. (A) Construction of simultaneously dual targeting fluorescent probe T-1 and cell membrane targeting and mitochondria targeting fluorescent probe CM-1 and Mito-1. (B) Calculated binding mode of T-1 to RNA. (C) Fluorescence spectra of T-1 (5 µM) in the presence of 0−9000 equiv. RNA. *λ*_ex_ = 425 nm. (D) Imaging of live cells (a), apoptotic cells (b) and dead cells (c) with T-1 (*λ*_ex_ = 405 nm, *λ*_ex_ = 600–650 nm); imaging of live cells (d), apoptotic cells (e) and dead cells (f) with CM-1(*λ*_ex_ = 470 nm, *λ*_ex_ = 500–550 nm); imaging of live cells (g), apoptotic cells (h) and dead cells (i) with Mito-1(*λ*_ex_ = 470 nm, *λ*_ex_ = 500–550 nm). (E) Illustration of simultaneous dual targeting fluorescent probe T-1 for monitoring the cell states (live cells, dead cells and apoptotic cells). Scale bar represents 10 µm.

To demonstrate that simultaneous dual targeting fluorescent probes can better distinguish multiple cellular states when compared to single-targeting probes. We compared probe T-1 with mitochondrial-targeting probe Mito-1 (Control 1) and membrane-targeting probe CM-1 (Control 2) (Fig. 4A). The molecular probe that we have developed with positive charge facilitates the monitoring of cellular apoptosis *via* loss of the mitochondrial membrane potential. Using these characteristics, we set out to monitor different cell states using T-1. The structure of the probe T-1 (shown in Fig. 2a) was optimized using Gaussian 09 software package, and molecular docking was performed with AutoDock 4.2 software, based on the crystal structure of RNA (RCSB Protein Data Bank ID: 1FJE). As shown in Fig. 4B and S14, the affinity of T-1 with RNA was 6.8 kcal mol^−1^, revealing that probe T-1 can bind to RNA which limits the intramolecular rotation resulting in enhanced fluorescence output for T-1. Next, to verify the theoretical results, RNA titration experiments were conducted to verify the response of probe T-1 toward RNA.

As shown in Fig. 4C, probe T-1 exhibited very weak absorption and emission spectra in buffer solution, which was significantly enhanced on the addition of RNA. The fluorescence emission intensity at 626 nm was significantly increased by about 25-fold, confirming that probe T-1 exhibits a strong binding affinity for RNA (0−9000 equiv). To rule out the potential interference of DNA, we investigated the changes in the fluorescence intensity of probe T-1 upon the addition of DNA using fluorescence spectroscopy (Fig. S15). The results showed that no significant alteration was observed in the fluorescence emission peak of the system, indicating that probe T-1 can specifically target and bind to RNA rather than DNA. In addition, the cell assays using CCK-8 (Fig. S6), confirmed that T-1 was nontoxic and suitable for live cell imaging. Therefore, to monitor cell viability, T-1, Mito-1, or CM-1 (5 µM) was incubated in HeLa cells for 30 min respectively. As shown in Fig. 4D(a), T-1 exclusively targets plasma and mitochondrial membranes. CM-1 labels the cell membranes (Fig. 4D(d)) and Mito-1 stains the mitochondria (Fig. 4D(g)). To evaluate the apoptosis in the cells, CCCP was added to the Petri dishes of each group and incubated for 5 min to induce depolarization of ΔΨm.^33^ Consistent with the expectations for the apoptotic cells, we observed that the fluorescence intensity of T-1 in the mitochondria was significantly decreased (Fig. 4D(b)). The cell membrane-anchoring probe CM-1 enters the cytoplasm and exhibits weak fluorescence response due to the permeability enhancement of the cell membrane during apoptosis (Fig. 4D(e)). Mito-1 is released from the mitochondria to the cytoplasm (Fig. 4D(g) and (h)) during apoptosis. Therefore, probe T-1 can distinguish between healthy cells and those undergoing early-stage apoptosis due to the membrane migration during apoptosis. Moreover, to distinguish between live and dead cells, fixed HeLa cells were incubated with T-1, Mito-1 or CM-1, respectively, and were then imaged. We observed that probe T-1 was distributed in both the nucleoli and cytoplasm (Fig. 4D(c)), while the controls Mito-1 and CM-1 were only distributed in the cytoplasm (Fig. 4D(f) and (i)). This indicates that probe T-1 can label RNA, so probe T-1 can clearly distinguish between living and dead cells. During cell death, the permeability of the cell nuclear membrane increases, and the probe enters the nucleus. Since the nucleoli have no membrane structure, T-1 can bind with RNA. By contrast, the single-targeting probes Mito-1 or CM-1 cannot be used to distinguish between live and dead cells.

Therefore, the dual targeting probe T-1 can judge healthy cells, the early apoptosis of cells and dead cells by migration between the target plasma and mitochondrial membranes (Fig. 4E). Interestingly, during the apoptotic process, T-1 just targets the plasma membrane because the mitochondrial membrane potential loses polarity. During cell death, T-1 binds to the RNA resulting in enhanced fluorescence. Significantly, the differentiation of the multiple cell states using fluorescent probes can aid in the evaluation of cellular physiological and pathological states and may help in the discovery of therapeutic targets.

### Simultaneous dual targeting photodynamic ablation of cancer cells

In recent years, single-anchoring photosensitizers (PSs) have been developed to induce the death of cancer cells through apoptosis and pyroptosis, including mitochondrial,^34–39^ endoplasmic reticulum^40–43^ and plasma membrane targeting systems.^44–46^ Nevertheless, photosensitizers are rarely applied to multiple biomembranes synergistically to rapidly enable malignant cell death, which restricts the efficiency of the photosensitizers, and results in unsatisfactory therapeutic outcomes. To overcome these limitations, simultaneous dual targeting PSs are required to overcome these obstacles.

**Fig. 5:**
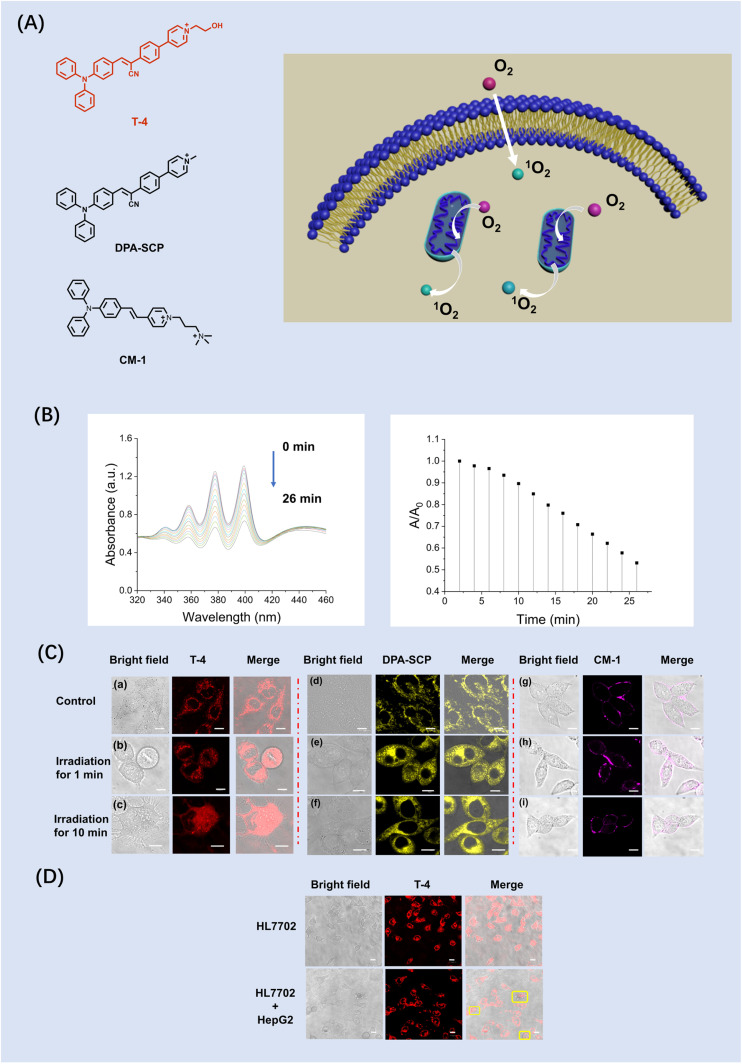
. Structure and imaging of dual-targeting photosensitizers. (A) The structure of T-4, DPA-SCP and CM-1 schematic illustration of AIEgen T-4 induced mitochondrial stress and the crosstalk between plasma membrane and mitochondria for cell apoptosis upon PDT. (B) Changes of UV-Vis spectra of ABDA (60 µM) in the presence of T-4 10 (µM) in water after continuous exposure for designated time intervals, where *A*_0_ is the initial absorbance maximum and *A* is the absorbance maximum of the samples at 378 nm before and after white light irradiation. (C) The HeLa cells were treated with T-4, **DPA-SCP** and CM-1 (10 µM) for 30 min (a, d and g) and then upon light irradiation for 1 min (b, e and h) and 10 min (c, f and i) under light irradiation at a power density with 40 mW cm^−2^ for 10 min. (D) Confocal images of mixture of **T-4** treated (30 min) HepG2 cells and HL-7702 cells after light irradiation for 10 min. The pyroptosis of HepG2 cells is highlighted by yellow boxes. Scale bars: 10 µm.

Therefore, we developed a new type of dual-targeting photosensitizer (T-4) to accelerate cancer cell ablation. Mitochondrial and plasma membrane dual targeting organic PSs are highly desirable for enhanced PDT efficiency. When T-4 is simultaneously located in the mitochondrial and cell membrane, the mitochondria and cell membrane generate substantial singlet oxygen (^1^O_2_) and ROS, respectively, upon light irradiation. The lipophilic group triphenylamine is an excellent electron donor and can not only enhance the push–pull of electrons, but also exhibits aggregation induced luminescence (Fig. 5A). Mitochondrial and cell membrane targeting, photosensitizer (T-4) can target cancer cells resulting in cancer cell oxeiptosis. T-4 was designed with an aggregation-induced emission (AIE) active triphenylamine derivative as the electron-donating group (EDG) and a cyano-pyridinium salt moiety as the electron-withdrawing group (EWG). The spectroscopic properties of T-4 in various solvents using UV-Vis spectroscopy and photoluminescence (PL) spectroscopy were evaluated (Fig. S6), T-4 exhibits a maximum absorption peak at 438 nm and a near-infrared (NIR) emission at 621 nm in aqueous solution, respectively. The Stokes shift was nearly 180 nm, which is favorable to effectively reduce the interference of the background signals, due to the stronger ICT effect caused by the cyano- and pyridinium EWGs. The fluorescence properties of T-4 were evaluated in a mixture of dimethyl sulfoxide (DMSO) and toluene. As shown in Fig. S16, T-4 exhibits almost no emission in pure DMSO solution. Since the T-4 structure is based on a donor–acceptor design, it can exhibit twisted intramolecular charge transfer (TICT). Hence, when T-4 is dissolved in DMSO, its emission is weakened by TICT and intramolecular motion results in nonradiative decay. Subsequently, toluene, a poor solvent, results in sharp emission enhancements due to the formation of T-4 aggregates, activating fluorescence by the restriction of intramolecular motion (RIM) process. To evaluate the influence of pH on T-4, we measured T-4 in PBS at various pH values (Fig. S17), which confirmed that T-4 exhibited good spectroscopic properties suitable for an intracellular microenvironment. In addition, the T-4 AIEgen exhibited good photostability compared with ICG (Indocyanine green) under laser irradiation for 30 min (Fig. S18). The *in vitro*^1^O_2_ generation capability of T-4 was evaluated under laser irradiation using 9,10-anthracenediyl-bis(methylene)dimalonic acid (ABDA) as the ^1^O_2_ indicator (Fig. 5B). ABDA can selectively react with ^1^O_2_, a typical ROS, resulting in a corresponding decrease in its absorbance. Upon light irradiation, the absorbance of ABDA in water exhibited an obvious change within 26 min in the presence of T-4 suggesting good ^1^O_2_ generation capability in water.

To verify that simultaneous dual targeting PSs are better than single targeting PSs, we synthesized a mitochondrion-anchoring photosensitizer **DPA-SCP** and plasma membrane-anchoring photosensitizer CM-1 as control. Firstly, we evaluated the suitability of the probe T-4 and **DPA-SCP** for biological systems by evaluating the dark and phototoxicity using a CCK-8 test kit (Fig. S19–S22), which confirmed that the cell viability was 84% with T-4 (50 µM). T-4, **DPA-SCP** and CM-1 (10 µM) were then incubated in the HeLa cells for confocal imaging at 37 °C under 5% CO_2_ for 30 min (Fig. 5C(a), (d) and (g)). Oxidative stress disrupts the cell membrane and leads to changes in cell morphology. To better understand the membrane targeting photodynamic cytocidal action, real-time observations of the cell morphological changes were conducted using T-4. Upon light irradiation for 1 min, the cells treated with T-4 exhibited the rupture of the cell membrane and mitochondrial swelling, leading to a change of cell morphology (Fig. 5C(b)). By contrast, the cells treated with the control **DPA-SCP** and CM-1 under the same conditions did not display the rupture of the cell membrane and mitochondrial swelling (Fig. 5C(e) and C(h)). To better observe the morphological changes of the cancer cells after photodynamic treatment, we incubated the cancer cells with T-4, and subjected them to light irradiation for 10 min (Fig. 5C(c)). Ruptured and intact plasma membranes are classically considered as the hallmarks of necrotic and apoptotic cell death, respectively. Significantly, the HeLa cells when incubated with mitochondrion-anchoring photosensitizer **DPA-SCP** and CM-1 exhibited no obvious morphological changes (Fig. 5C(f) and (i)). After light irradiation for 10 min, only the plasma membrane of the cancer cells (HepG2) were morphologically ruptured (Fig. 5D). Taken together, these results confirm that the simultaneous dual targeting photosensitizer T-4 exhibited better photodynamic efficiency than the control **DPA-SCP** and CM-1.

## Conclusion

In summary, we have proposed a new rational strategy to establish a single molecular group for the simultaneous targeting of two distinct organelles. Based on this strategy, we designed and constructed a novel dual targeting group, which was composed of a positively charged pyridinium unit with a hydroxyl group, achieving the simultaneous targeting of the mitochondria and plasma membrane. The results of the experimental and theoretical calculations confirmed that our rational strategy provides an unprecedented dual targeting group (4-(4-(cyanomethyl)phenyl)-1-(2-hydroxyethyl)pyridin-1-ium) for labeling lipid rafts and mitochondria. By exploiting the new dual targeting group we further developed two dual targeting molecular tools T-1 and T-4. Using targeted migration between the organelles, the dual targeting florescent probe T-1 can determine the various cell states and facilitate monitoring the variation of specific physicochemical properties of the organelles during the transition from the healthy state to early apoptosis and dead states. Significantly, T-1 may help in the discovery of therapeutic targets. Moreover, T-4, a dual targeting photosensitizer, could rupture the plasma membrane and expand the mitochondria to enhance the photosensitizers efficiency in accelerating cancer cells necrosis. We believe that the strategy used in this research is valuable for advancing the field of organelle targeting research. In addition, we anticipate that such dual targeting groups will provide inspiration for researchers in developing dual targeting functional tools, to help resolve difficult scientific puzzles in multiple fields of research.

## Author contributions

W. L. conceived the idea and designed the experiments with Y. L.; Y. L., Y. Y., Y. Z., T. J discussed and analyzed the results. Y. L. performed the quantum chemical calculations, photophysical measurements, and imaging cells. Y. L. and Y. Y. synthesized and validated the majority of T-1 derivatives. Y. L. W. L., and T. J wrote the paper. All the authors discussed the results and commented on the manuscript.

## Conflicts of interest

There are no conflicts to declare.

## Supplementary Material

SC-OLF-D5SC06276D-s001

## Data Availability

All relevant data is presented in the paper and supplementary information (SI). Supplementary information: experimental details including synthesis, the characterization of the compounds, absorption and fluorescence spectroscopic data, and imaging in vivo. See DOI: https://doi.org/10.1039/d5sc06276d.
