# Supplementary material for: A molecular scaffold for concurrent targeting of plasma and mitochondrial membranes
Source: Chem Sci. 2026 Jan 16;17(9):4507–17. doi: 10.1039/d5sc06276d (PMC12809707; doi:10.1039/d5sc06276d)
Supplement: SC-017-D5SC06276D-s001 [file SC-017-D5SC06276D-s001.pdf]

Supplementary Information (SI) for Chemical Science.
This journal is © The Royal Society of Chemistry 2026

Supporting Information for

A Molecular Scaffold for Concurrent Targeting of Plasma and Mitochondrial Membranes

Youbo Lai [1], Yi Yang [1], Yuping Zhao [1], Tony D. James [2,3,*] & Weiying Lin[1,*]

[1] *Institute of Optical Materials and Chemical Biology, Guangxi Key Laboratory of Electrochemical Energy Materials, School of Chemistry and Chemical Engineering, Guangxi University, Nanning, Guangxi 530004, P. R. China.*

[2] *Department of Chemistry, University of Bath, Bath, BA2 7AY, UK,*

[3] *School of Chemistry and Chemical Engineering, Henan Normal University, Xinxiang 453007, P. R. China*

E-mail: weiyinglin2013@163.com；t.d.james@bath.ac.uk

Table of contents

Pages

Experimental S4

Materials and instruments... S4

Methods.. S4

Synthesis and characterization of T-1 to T-5.. S7

Synthesis and characterization of S-1, S-1, CM-1, Mito-1 and DPA-SCP S8

Figure. S3. .... S12

Figure S4. .... S13

Figure S5. .... S13

Figure S6 .... S14

Figure S7 .... S14

Figure S8 .... S15

Figure S9 .... S16

Figure S10 .... S16

Figure S11 .... S17

Figure S12 .... S17

Figure S13 .... S17

Figure S14 .... S18

Figure S15 .... S19

Figure S16 .... S20

Figure S17 .... S20

Figure S18 .... S17

Figure S19. .... S18

Figure. S20 .... S18

Figure. S21 .... S19

Figure. S22 .... S19

Figure. S23 .... S20

Figure. S24 .... S20

Figure. S25 .... S21

Figure. S26 .... S21
Figure. S27 .... S22
Figure. S28 .... S27
Figure. S29 .... S23
Figure. S30 .... S23
Figure. S31 .... S24
Figure. S32 .... S24
Figure. S33 .... S25
Figure. S34 .... S25
Figure. S35 .... S26
Figure. S36 .... S26
Figure. S37 .... S27
Figure. S38 .... S27
Figure. S39 .... S28
Figure. S40 .... S28
Figure. S41 .... S29
Figure. S42 .... S29
Figure. S43 .... S33
References S34

Experimental

Materials and instruments.

All reagents were obtained from commercial suppliers without further purification. All experiments used ultra-pure water. Solvents were purified by standard methods prior to use. The pH measurements were performed using a PHS-3E pH meter. CCK-8 was purchased from Bide Pharmatech Ltd. Fluorescence imaging experiments were performed using HITACHI F47000 fluorescence spectrophotometer. TLC analysis was carried out on silica gel plates, and column chromatography was conducted over silica gel (mesh 200-300); purchased from the Qingdao Ocean Chemicals. ^{1}H and ^{13}C NMR spectra were measured using a Varian Unity 500 spectrometer. High resolution mass spectrometric (HRMS) analyses were measured on Aglient 7250& JEOL-JMS-T100LP.

Methods

Cell culture and cytotoxicity assay

HeLa, HL-7702 and HepG2 cells were adhered to a glass bottom dish with 1 mL cultured in Dulbecco's modified Eagle's medium (DMEM) supplemented with 10 % fetal bovine serum (FBS), 100 U/mL penicillin and 100 μg/mL streptomycin under an atmosphere of 5 % CO_2 at 37 °C. The fluorescence imaging of cells was performed using a Leica TCS SP8 CARS confocal microscope. HeLa cells were seeded in a plastic-bottomed 96-well plate (5×10^3 cells/ well) at a density of 7.6×10^4 cells per well. After 24 hours, the medium was aspirated and replaced with fresh medium containing various concentrations of probes (adjusted by diluting with 10 mM DMSO stock solution ≤ 0.1% (v/v)). After incubation for 24 h, the medium was aspirated and replaced with media containing 5% Cell Counting (cell-counting Kit-8) at 37 °C for 4 h. After further incubation probes (final concentration 0, 1, 2, 5, 10, 50, 100, and 150 μM) were added for 1 h, the absorbance at 405 nm was measured using a microplate reader (TransGen Biotechnology, China) to determine the cell viability. In the cytotoxicity assay, the group with a probe concentration of 0 μM (i.e., the control group) was treated with dimethyl sulfoxide (DMSO) alone without the addition of probes. Notably, the final concentration of DMSO in all experimental groups was strictly controlled at ≤0.1% (v/v) to eliminate potential solvent-induced cytotoxicity. The cellular viability of each group was

determined by defining the absorbance of the control group to be 100% viability. The assays were performed six times for each concentration.

Ultraviolet-visible (UV-vis) absorption and fluorescence spectroscopy

UV-vis absorption spectra were obtained on a Shimadzu UV-2700 spectrophotometer, and fluorescence spectra were measured on a HITACHI F4700 fluorescence spectrophotometer. The fluorescence imaging of cells was performed using a Leica TCS SP8 CARS confocal microscope. The photomultiplier voltage was 500 V.

Molecular simulation

The geometrical structure and frontier orbitals of probes was obtained with Gaussian 09 software, by means of the sequential optimization with the basic set of b3lyp/3-21g, and b3lyp/6-31g, respectively. The binding mode of **T-1** to RNA was calculated via AutoDock 4.2 software. For AutoDock calculations, number of GA runs was set as 50, Maximum number of evals was set as 25000000, and the other parameters were set as the default ones without changes.

Colocalization Experiments

The commercially available fluorescent probe MitoTracker Deep-Red FM(MTDR), CellTracker DiO and Dil were used for colocalization experiments. The probe **T-1** (1 μM), MTDR (200 nM), DiO (1 μM) and Dil (1 μM) were used to coincubate HeLa cells for 30 min, and then cell images were directly acquired with the Leica TCS SP8 CARS confocal microscope. The fluorescent signal of DiO in blue channel (λ_{ex} = 480 nm, λ_{ex}= 501-530 nm), DiI in the yellow channel (λ_{ex} = 553 nm, λ_{ex}= 560-600 nm), **T-1** in yellow channel (λ_{ex} = 405 nm, λex= 600-650 nm) and MTDR in red channel (λ_{ex} = 644 nm, λ_{ex}= 660-680 nm). The Pearson's colocalization coefficient and colocalization scatter plot were obtained by TCS SP8 CARS confocal software.

CCCP Treated Experiments

Carbonyl cyanide 3-chlorophenylhydrazone (CCCP) treated cells were used to determine the response of **T-1** to ΔΨm. Cells in a glass bottom culture dish were preincubated with **T-1** (1 μM) for 30 min and placed under the microscope, and then 2 μL of CCCP solution (5 mM in DMSO) was

premixed with 100 μL of culture medium and dropped into the culture dish to induce depolarization of ΔΨm.

Synthesis and characterization of T-1 to T-5

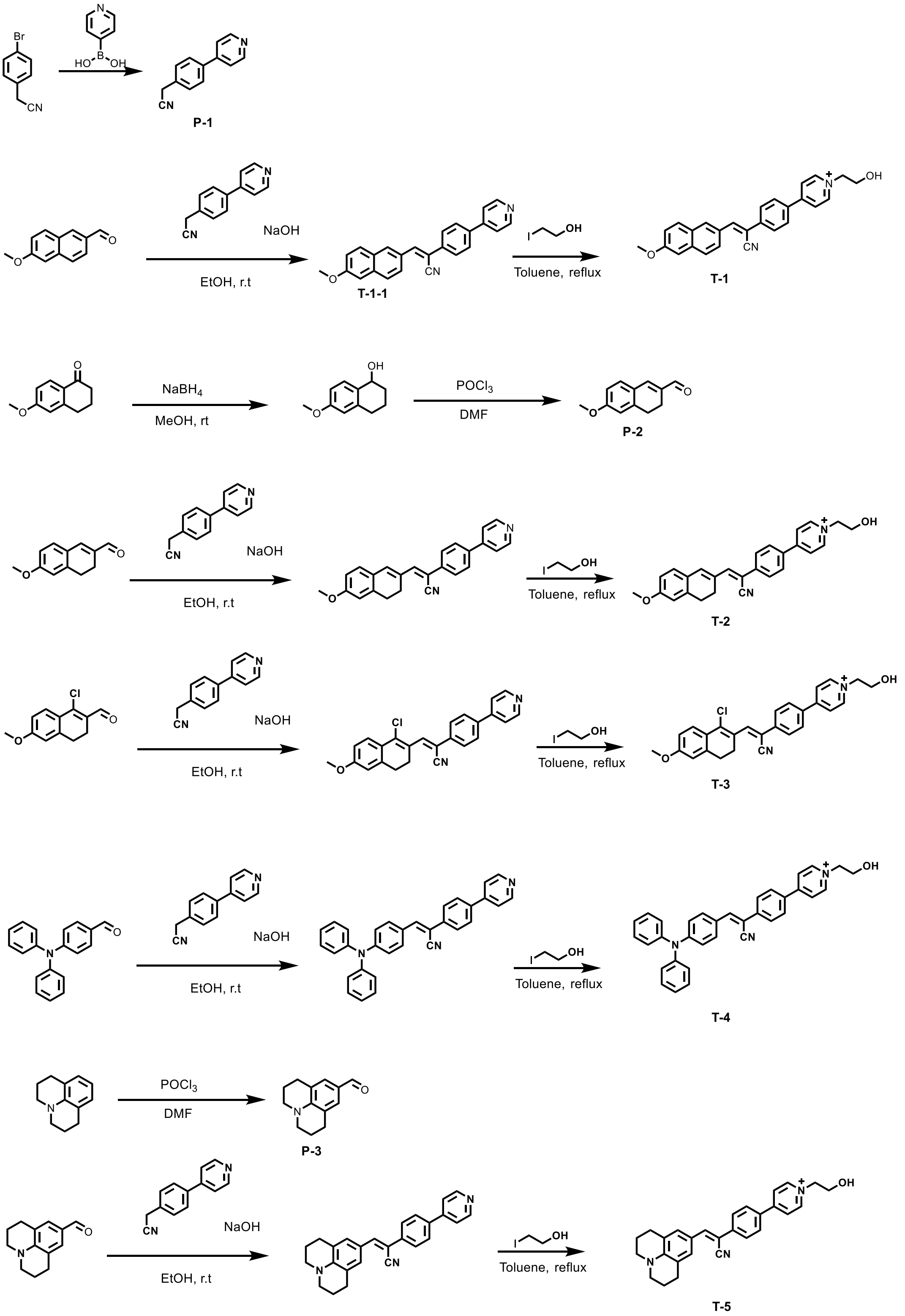

Figure. S1 Synthesis of probes T-1 to T-5.

Synthesis of P-1

2-(4-bromophenyl)acetonitrile (1.0 equiv), 2-(4-(pyridin-4-yl)phenyl)acetonitrile (2.0 equiv) and $Pd(PPh_3)_4$ (320 mg) were dissolved in acetonitrile (28 mL) and 2 M Sodium carbonate solution (5.3 mL) then stirred for 24 h at 80 °C. The mixture was then cooled to room temperature and the white solid was collected by filtration. The filtrate was then diluted with ethyl acetate and extracted with saturated brine (3 times), dried over Na_2SO_4 and concentrated under reduced pressure. The residue was purification by column chromatography on silica gel with an eluent of DCM: MeOH=50: 1 to afford P-1 in 180 mg, 0.94 mmol 80 % yield. ^{1}H NMR (500 MHz, $CDCl_3$) δ 8.68 (dd, J = 4.6, 1.6 Hz, 2H), 7.66 (d, J = 8.3 Hz, 2H), 7.51 (dd, J = 4.5, 1.6 Hz, 2H), 7.47 (d, J = 8.3 Hz, 1H), 3.82 (s, 2H).

Synthesis of T-1

P-1 (388.5 mg) was dissolved in 10 ml MeOH and 5 drops of piperidine were added. Then, 6-methoxy-2-naphthaldehyde 402.5 mg was added and the mixture was stirred for 6 h at 80 °C. The mixture was then cooled to room temperature and the product filtered to give (T-1-1) 610 mg, 1.68 mmol 84 % yield. ^{1}H NMR (500 MHz, $CDCl_3$) δ 8.70 (dd, J = 4.6, 1.4 Hz, 2H), 8.25 (s, 1H), 8.12 (dd, J = 8.7, 1.7 Hz, 1H), 7.82 (td, J = 8.6, 5.4 Hz, 4H), 7.76 – 7.69 (m, 3H), 7.55 (dd, J = 4.6, 1.6 Hz, 2H), 7.20 (dd, J = 8.9, 2.5 Hz, 1H), 7.16 (d, J = 2.3 Hz, 1H), 3.96 (s, 3H). T-1-1 379 mg and 2-iodoethan-1-ol 125 mg were dissolved in 10 mL of toluene and stirred for 6 h at 120 °C. The mixture was then cooled to room temperature and the solid filtered. The crude product was purified by silica-gel column chromatography using DCM/ MeOH (v/v = 40:1) as eluent to furnish an orange solid as product **T-1**(310 mg, 0.76 mmol, 76% yield). ^{1}H NMR (500 MHz, DMSO) δ 9.07 (d, J = 7.0 Hz, 2H), 8.62 (d, J = 7.0 Hz, 2H), 8.41 (d, J = 24.2 Hz, 2H), 8.27 (d, J = 8.6 Hz, 2H), 8.21 – 8.16 (m, 1H), 8.07 (d, J = 8.6 Hz, 2H), 7.97 (dd, J = 22.8, 8.8 Hz, 2H), 7.44 (d, J = 2.4 Hz, 1H), 5.28 (s, 1H), 4.71 – 4.64 (m, 2H), 3.94 (s, 5H).

Synthesis of P-2

6-methoxy-3,4-dihydronaphthalen-1(2H)-one (528.7 mg, 1 eq) was dissolved in 20 mL MeOH.

Then $NaBH_4$ (567.5 mg, 5eq) was added in three portions at 0 °C under a nitrogen atmosphere. The mixture was then heated to room temperature for 3h with stirring. The precipitate was filtered by suction filtration. After solvent evaporation, the crude product was diluted with DCM and the crude product was used for the next step without further purification. Phosphorus oxychloride (3 mL) was added dropwise at 0 °C to DMF (10 mL). The mixture was stirred at 0 °C for 1h and then at room temperature for 30 min. To the resulting solution 6-methoxy-1,2,3,4-tetrahydronaphthalen-1-ol was then added in one portion. The reaction mixture was heated to 100 °C for 3h. After cooling, the mixture was poured into ice water (40 mL) which contained sodium acetate, and then 5% NaOH solution was added to adjust the pH to 12. The light brown solid product was then collected (390 mg, 2.07 mmol, 63 %) by filtration, washed with water, and dried. ^1H NMR (500 MHz, DMSO) δ 9.57 (s, 1H), 7.48 (s, 1H), 7.36 (d, J = 8.0 Hz, 1H), 6.89 – 6.82 (m, 2H), 3.80 (s, 3H), 2.80 (t, J = 8.3 Hz, 2H), 2.42 – 2.35 (m, 2H).

Synthesis of T-2

2-(4-bromophenyl)acetonitrile (1.0 equiv), P-2 (402.5 mg) and $Pd(PPh_3)_4$ (320 mg) were dissolved in acetonitrile (28 mL) and 2 M Sodium carbonate solution (5.3 mL) then stirred for 24 h at 80 °C. Then, the mixture was cooled to room temperature and the white solid removed by filtration. The filtrate was diluted with ethyl acetate and extracted using saturated brine (3 times) and dried over Na_2SO_4 and the solution was concentrated under reduced pressure. Purification by column chromatography on silica gel with eluent DCM: MeOH=50: 1 to afford the desired product (210 mg, 0.51 mmol, 45 % yield). ^1H NMR (500 MHz, DMSO) δ 9.04 (d, J = 6.8 Hz, 2H), 8.59 (d, J = 6.9 Hz, 2H), 8.22 (d, J = 8.6 Hz, 2H), 7.99 – 7.80 (m, 3H), 7.32 – 7.15 (m, 2H), 6.94 – 6.76 (m, 2H), 5.28 (s, 1H), 4.74 – 4.60 (m, 2H), 3.98 – 3.85 (m, 2H), 3.80 (s, 3H), 2.94 (dd, J = 11.6, 5.6 Hz, 4H). ^{13}C NMR (126 MHz, $CDCl_3$) δ 160.73 (s), 155.22 (s), 147.81 (s), 145.26 (s), 139.10 (s), 138.52 (s), 138.05 (s), 133.90 (s), 132.18 (s), 130.83 (s), 129.50 (s), 128.17 (s), 126.14 (s), 125.05 (s), 120.76 (s), 113.56 (s), 112.04 (s), 107.90 (s), 63.02 (s), 60.64 (s), 55.44 (s), 28.30 (s), 25.51 (s).

Synthesis of T-3, T-4 and T-5

T-3, T-4 and T-5 were synthesized according to the same method used for **T-2**.

T-3. ^1H NMR (500 MHz, DMSO) δ 9.05 (d, J = 6.9 Hz, 2H), 8.56 (dd, J = 12.3, 7.0 Hz, 2H), 8.22

(d, J = 8.6 Hz, 2H), 8.10 (s, 1H), 7.94 (d, J = 8.6 Hz, 2H), 7.64 (d, J = 9.1 Hz, 1H), 6.96 – 6.86 (m, 2H), 5.29 – 5.23 (m, 1H), 4.73 – 4.59 (m, 2H), 3.90 (dd, J = 9.9, 5.1 Hz, 2H), 3.83 (s, 3H), 3.07 (dd, J = 8.9, 6.6 Hz, 2H), 2.94 (t, J = 7.8 Hz, 2H). ^{13}C NMR (126 MHz, DMSO) δ 161.49 (s), 153.98 (s), 145.76 (s), 141.90 (s), 140.20 (s), 138.15 (s), 137.32 (s), 134.38 (s), 129.51 (s), 128.37 (s), 128.14 (s), 127.27 (s), 125.07 (s), 124.58 (s), 117.80 (s), 113.74 (s), 113.00 (s), 109.56 (s), 62.82 (s), 60.55 (s), 55.99 (s), 27.60 (s), 26.72 (s).

T-4. ^{1}H NMR (500 MHz, DMSO) δ 9.04 (d, J = 7.0 Hz, 2H), 8.59 (d, J = 7.0 Hz, 2H), 8.23 (d, J = 8.7 Hz, 2H), 7.99 (d, J = 8.7 Hz, 2H), 7.93 (d, J = 9.0 Hz, 2H), 7.42 (dd, J = 8.3, 7.6 Hz, 4H), 7.24 – 7.16 (m, 6H), 6.97 (d, J = 8.9 Hz, 2H), 5.27 (t, J = 5.3 Hz, 1H), 4.69 – 4.64 (m, 2H), 3.90 (dd, J = 9.9, 5.2 Hz, 2H). ^{13}C NMR (126 MHz, DMSO) δ 154.05 (s), 150.49 (s), 146.30 (s), 145.69 (s), 144.39 (s), 138.29 (s), 133.59 (s), 131.75 (s), 130.41 (s), 129.34 (s), 126.81 (s), 126.29 (s), 125.43 (s), 124.42 (s), 120.01 (s), 105.12 (s), 62.76 (s), 60.56 (s).

T-5. ^{1}H NMR (500 MHz, DMSO) δ 9.01 (d, J = 7.0 Hz, 2H), 8.57 (d, J = 7.0 Hz, 2H), 8.19 (d, J = 8.7 Hz, 2H), 7.95 – 7.83 (m, 3H), 7.54 (s, 2H), 5.27 (s, 1H), 4.69 – 4.61 (m, 2H), 3.90 (dd, J = 9.9, 5.2 Hz, 2H), 3.32 – 3.27 (m, 4H), 2.71 (t, J = 6.2 Hz, 4H), 1.95 – 1.84 (m, 4H). ^{13}C NMR (126 MHz, DMSO) δ 154.11 (s), 146.06 (s), 145.56 (s), 144.92 (s), 139.47 (s), 132.25 (s), 129.87 (s), 129.24 (s), 125.96 (s), 124.06 (s), 120.81 (s), 119.86 (d, J = 19.3 Hz), 99.06 (s), 62.65 (s), 60.56 (s), 49.74 (s), 27.66 (s), 21.32 (s).

Synthesis and characterization of S-1, S-1, CM-1, Mito-1 and DPA-SCP

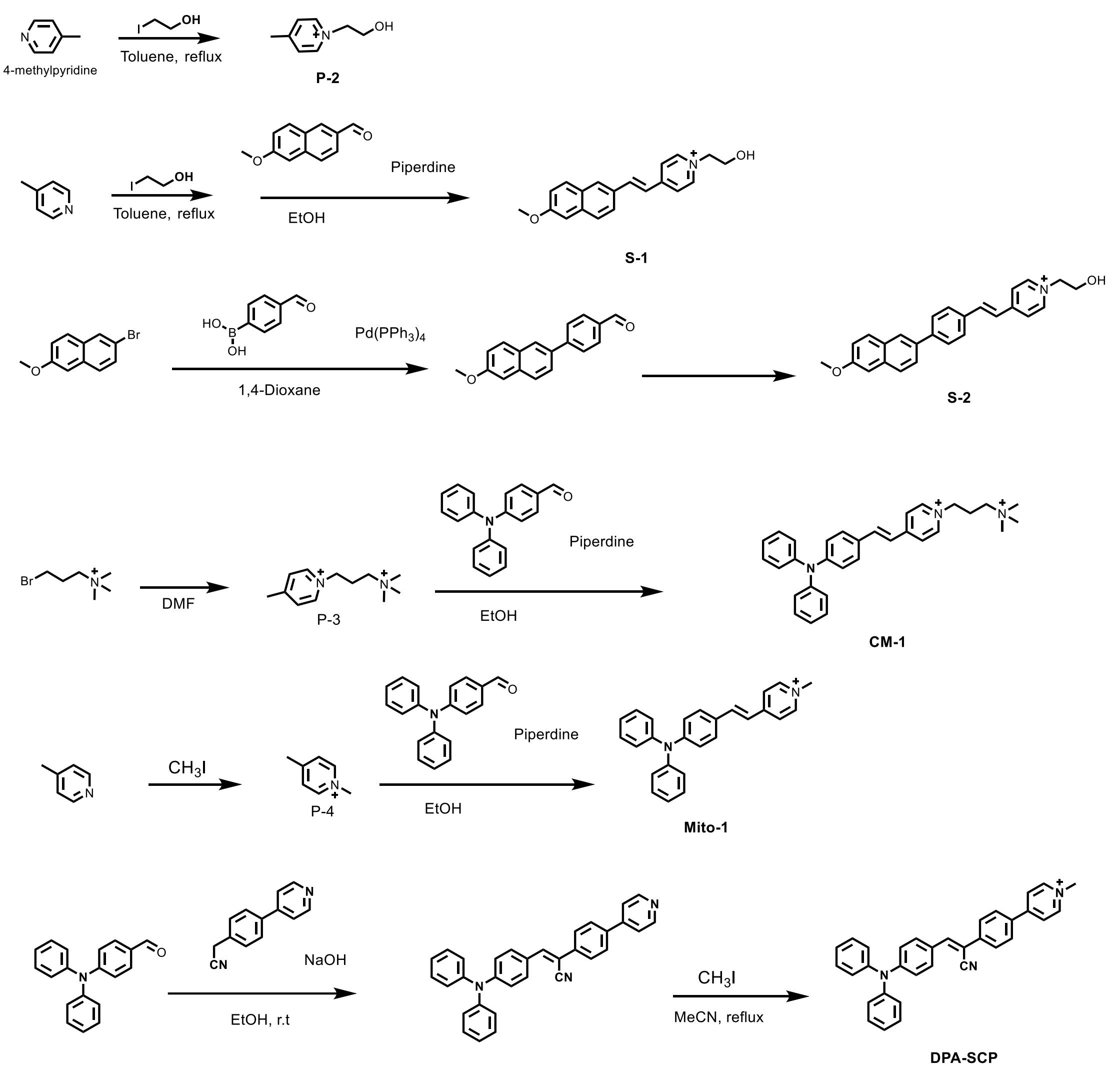

Figure. S2 Synthesis of compounds S-1, S-1, CM-1, Mito-1 and DPA-SCP

Synthesis of **S-1**

4-methylpyridine (1.5 mL) and 2-iodoethan-1-ol (2 mL) were dissolved in 10 mL toluene and the reaction mixture was heated to 110 °C for 6h. After the reaction was cooled to room temperature. The solvent was removed under reduced pressure to provide a residue which was purified by column chromatography for use in the next reaction. 6-methoxy-2-naphthaldehyde (186.2 mg, 1 mmol) and 200 μL piperdine were dissolved in 10 mL ethanol at room temperature for 10 min. Then **P-2** (138.2 mg, 1 mmol) in ethanol was added to the reaction mixture. The mixture was stirred at 80 °C for 5h. After the reaction was complete, the mixture was concentrated in vacuo to provide a residue which was purified by column chromatography to yield S-1 as a yellow solid (210 mg, 0.69 mmol, 69 %

yield). ^{1}H NMR (500 MHz, DMSO) δ 8.87 (d, J = 6.9 Hz, 2H), 8.27 (d, J = 6.9 Hz, 2H), 8.19 – 8.12 (m, 2H), 7.95 – 7.89 (m, 3H), 7.62 (d, J = 16.3 Hz, 1H), 7.39 (t, J = 4.3 Hz, 1H), 7.24 (dd, J = 8.9, 2.5 Hz, 1H), 5.24 (t, J = 5.3 Hz, 1H), 4.56 (dd, J = 12.5, 7.4 Hz, 2H), 3.93 – 3.85 (m, 5H).

Synthesis of **S-2**

474.2mg (2.02 mmol) of 2-bromo-6-methoxynaphthalene and 311.9 mg (2.07 mmol) (4-formylphenyl) boronic acid were dissolved in 15 mL methanol until they were completely dissolved. Then 300 mg K2CO3 and 100 mg catalyst Pd(PPh3)4 were added to the mixture stirred at 65 ℃ under an nitrogen atmosphere for 8h. The mixture was concentrated in vacuo to provide a residue that was used directly in the next reaction.

P-2 (90.2 mg, 1.87 mmol), 100 µL piperidine and 4-(6-methoxynaphthalen-2-yl)benzaldehyde (520 mg, 1.98 mmol) were dissolved 15 ml in ethanol in three neck flasks until completely dissolved under nitrogen protection. The mixture was stirred at 80 °C for 10 h. After completion of the reaction, the mixture was concentrated under reduced pressure to provide a residue which was purified by column chromatography to yield 63 % S-2 (480 mg, 1.26 mmol) as an orange solid. 1H NMR (500 MHz, DMSO) δ 8.90 (d, J = 6.5 Hz, 2H), 8.29 (d, J = 6.4 Hz, 3H), 8.11 (d, J = 16.3 Hz, 1H), 7.92 (ddd, J = 11.6, 10.6, 6.0 Hz, 7H), 7.62 (d, J = 16.2 Hz, 1H), 7.37 (s, 1H), 7.21 (dd, J = 6.5, 2.4 Hz, 1H), 5.25 (s, 1H), 4.58 (s, 2H), 3.91 (d, J = 5.5 Hz, 5H).

Synthesis of **CM-1**

Compound **P-2** was synthesized according to previous literature.[1] **P-3** (354 mg, 1.58 mmol) and 4-(diphenylamino)benzaldehyde (273 mg, 1 mmol) were dissolved in 10 mL EtOH. Then 100 µL piperidine was added to the mixture. The mixture was stirred at 80 ℃ for 24 h. After the reaction was complete, the mixture was concentrated in vacuo to give a residue which was purified by column chromatography to yield CM-1 in 89 % yield (398 mg, 0.89 mmol) as an orange solid. ^{1}H NMR (500 MHz, DMSO) δ 8.94 (d, J = 6.9 Hz, 2H), 8.23 (d, J = 6.9 Hz, 2H), 8.02 (d, J = 16.2 Hz, 1H), 7.64 (d, J = 8.8 Hz, 2H), 7.42 – 7.32 (m, 5H), 7.15 (ddd, J = 9.4, 7.9, 0.9 Hz, 6H), 6.95 (d, J = 8.8 Hz, 2H), 4.56 (t, J = 7.3 Hz, 2H), 3.45 – 3.40 (m, 2H), 3.10 (s, 9H), 2.46 – 2.38 (m, 2H).

Synthesis of **Mito-1**

Mito-1. ^{1}H NMR (500 MHz, DMSO) δ 8.79 (d, J = 6.9 Hz, 2H), 8.15 (d, J = 6.9 Hz, 2H), 7.95 (d, J = 16.2 Hz, 1H), 7.63 (d, J = 8.8 Hz, 2H), 7.39 (dd, J = 8.3, 7.5 Hz, 4H), 7.31 (d, J = 16.2 Hz, 1H), 7.19 – 7.09 (m, 6H), 6.95 (d, J = 8.8 Hz, 2H), 4.22 (s, 3H).

Synthesis of **DPA-SCP**

The Synthesis of **DPA-SCP** according to previous literature.[2]

DPA-SCP ^{1}H NMR (500 MHz, DMSO) δ 9.02 (d, J = 7.0 Hz, 2H), 8.54 (t, J = 10.0 Hz, 2H), 8.21 (d, J = 8.7 Hz, 2H), 8.14 (d, J = 3.4 Hz, 1H), 7.98 (d, J = 8.7 Hz, 2H), 7.92 (d, J = 9.0 Hz, 2H), 7.44 – 7.36 (m, 4H), 7.24 – 7.12 (m, 6H), 7.00 – 6.94 (m, 2H), 4.33 (s, 3H).Compound **1** (228 mg, 1.0 mmol) and 1-ethyl-2-methylquinolin-1-ium (299 mg, 1.0 mmol) were dissolved in EtOH (10 mL). Then piperidine (0.3 mL) was added with stirring and the mixture was heated at reflux for 16 h. The obtained solution was concentrated and the crude product was purified by column chromatography (CH_2Cl_2: CH_3OH = 30: 1) to afford a blue solid (compound **2**, 55 mg, yield 14%).

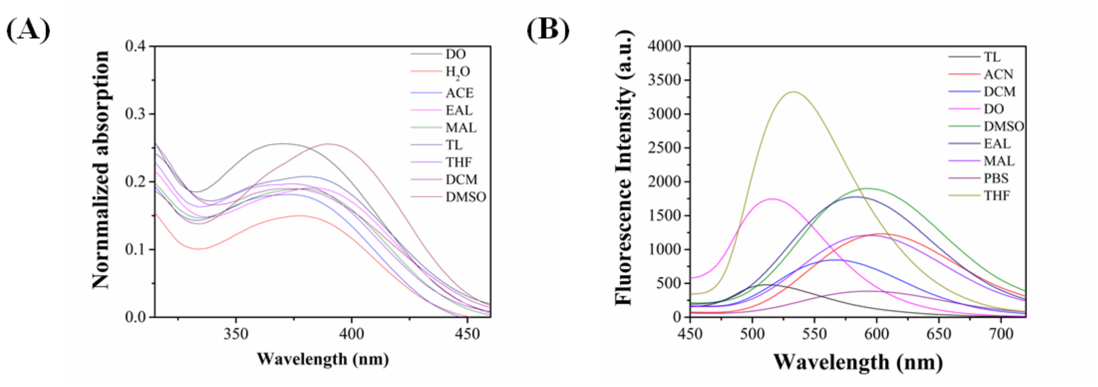

Figure. S3. The absorption and emission spectra of **T-1** in 1,4-Dioxane, ethanol, water, acetone, methanol, toluene, tetrahydrofuran, dichloromethane and dimethyl sulfoxide**.**

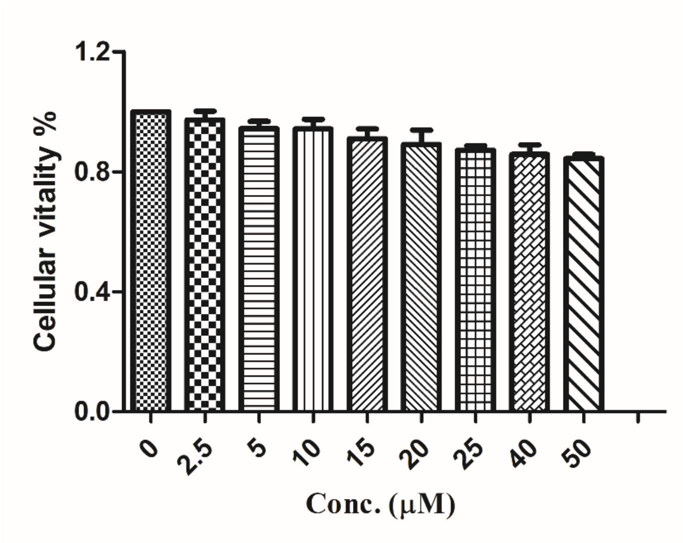

Figure S4. The cell viability of HeLa cells incubated with 2.5-50 μM of **S-1** for 24 h.

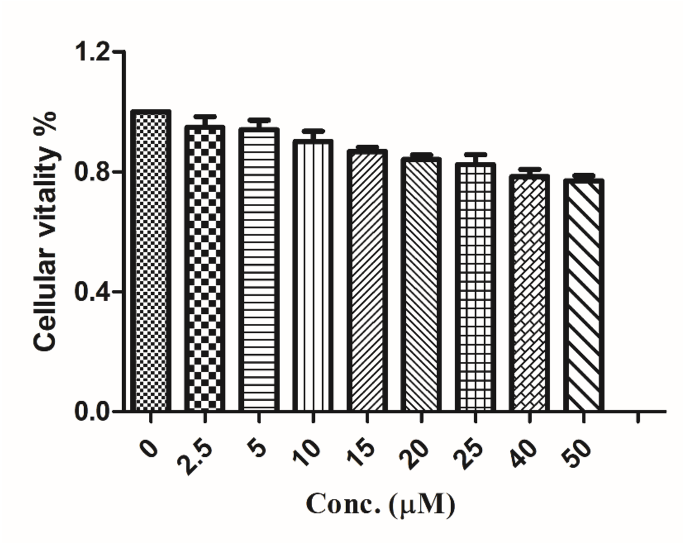

Figure S5. The cell viability of HeLa cells incubated with 2.5-50 μM of **S-2** for 24 h.

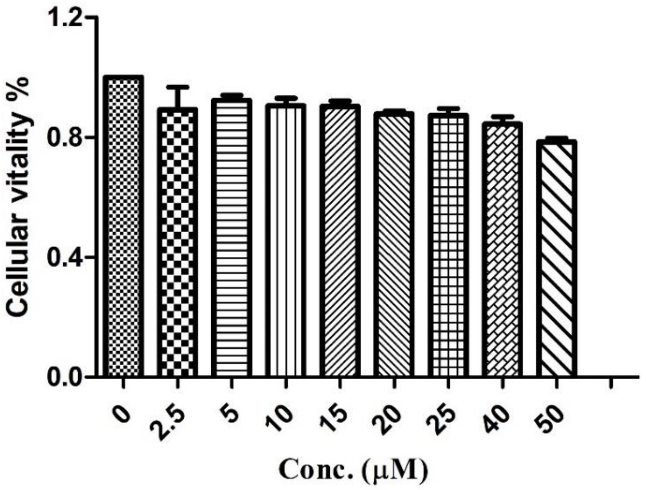

Figure S6. The cell viability of HeLa cells incubated with 2.5-50 μM of **T-1** for 24 h.

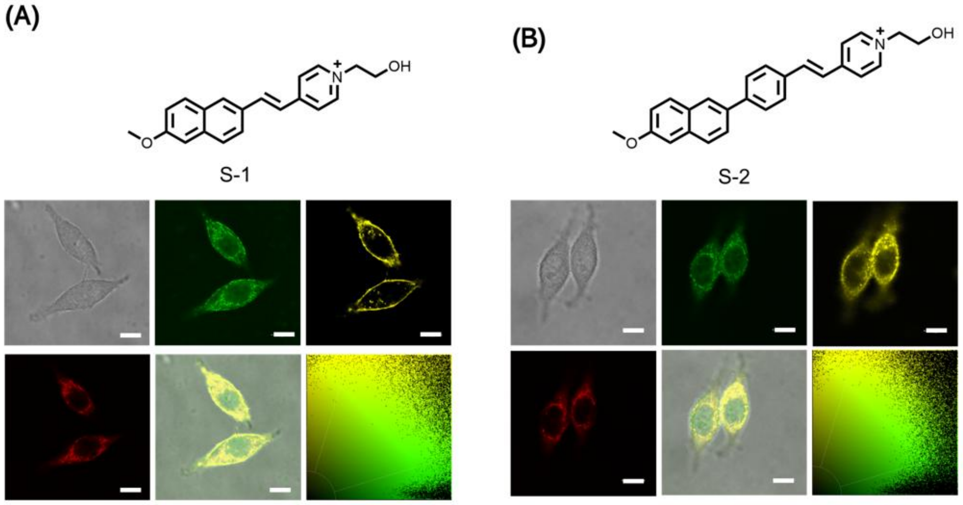

Figure S7. (A) Colocalization images of HeLa cells with **S-1** in green channel (λ_{ex} = 405 nm, λ_{ex}= 550-600 nm), DiI (λ_{ex} = 553 nm, λ_{ex}= 560-600 nm) in yellow channel and Mito Tracker Deep Red FM in red channel (λ_{ex} = 644 nm, λ_{ex}= 660-680 nm); (B) Colocalization images of HeLa cell with **S-2** in green channel (λ_{ex} = 405 nm, λ_{ex}= 600-650 nm), DiI (λ_{ex} = 553 nm, λ_{ex}= 560-600 nm) in yellow channel and MitoTracker™ Deep Red FM in red channel (λ_{ex} = 644 nm, λ_{ex}= 660-680 nm) Scale bars: 10 μm.

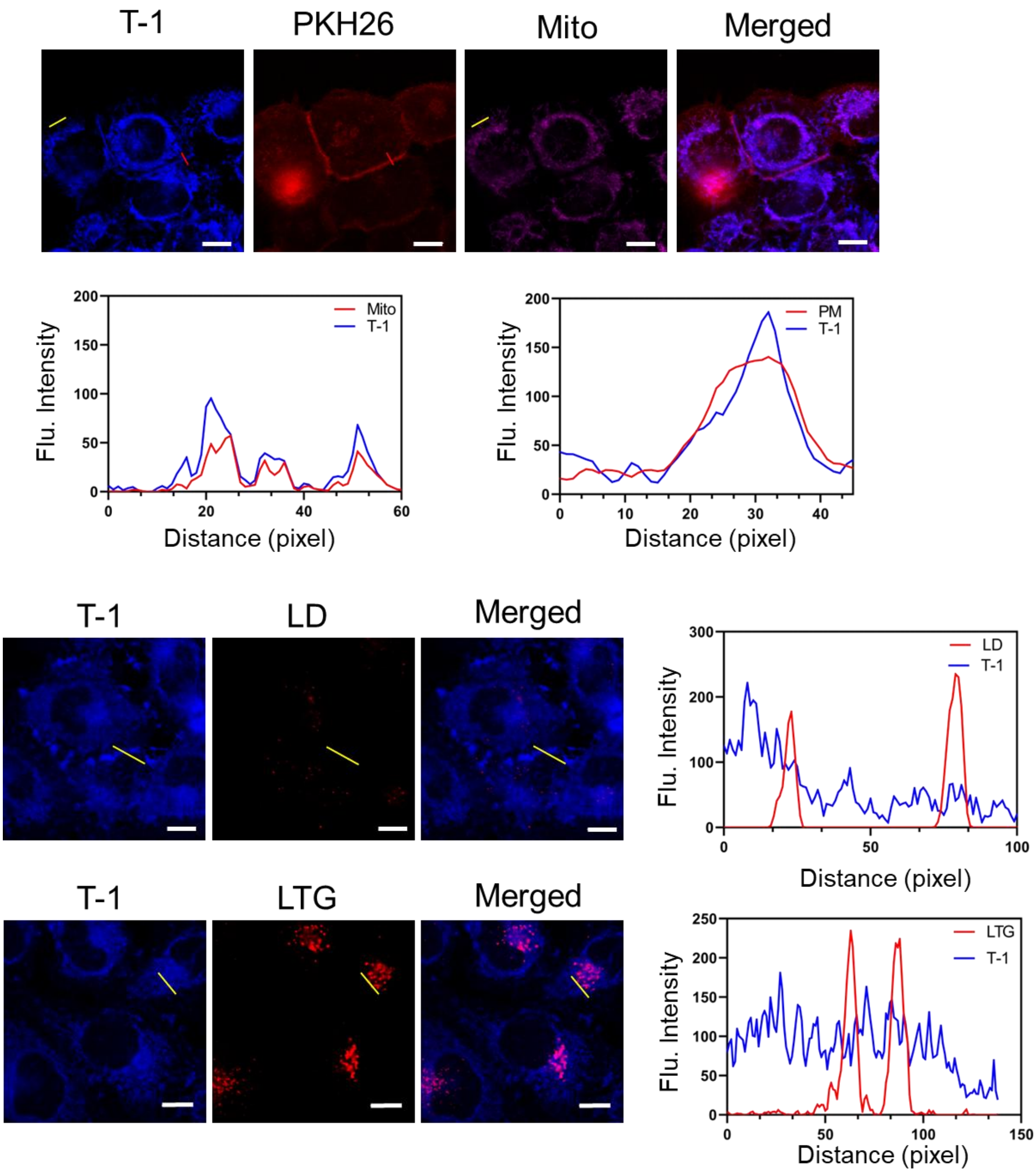

Figure S8. Colocalization images of HepG2 cells stained by **T-1** (1 μM, λ_{ex} =405 nm/λ_{em}=600-650 nm) and different commercial probes including Mito (500 nM, λ_{ex} =633 nm/λ_{em}=640-660 nm), PKH26 (1 μM, λ_{ex} =550 nm/λ_{em}=560- 600 nm), LTG (100 nM, λ_{ex} =504 nm/λ_{em}=510-560 nm), and BODIPY 493 (500 nM, λ_{ex} =493 nm/λ_{em}=503-553 nm). The inset diagrams represent the linear analysis of the selected regions of the HepG2 cells after staining with **T-1** and different probes. Scale bar: 10 μm.

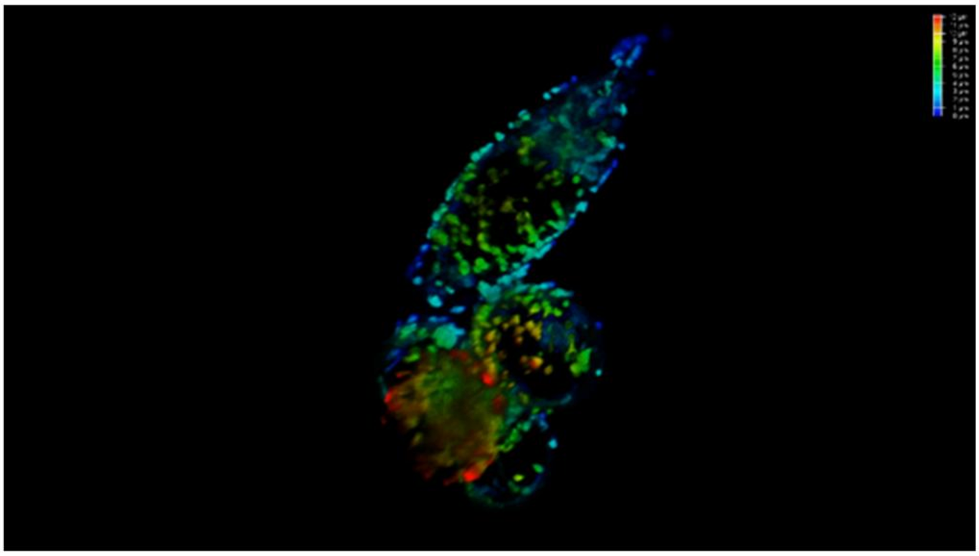

Figure S9. The imaging of HeLa with **T-1** in 3D mode. Scale bars: 10 μm.

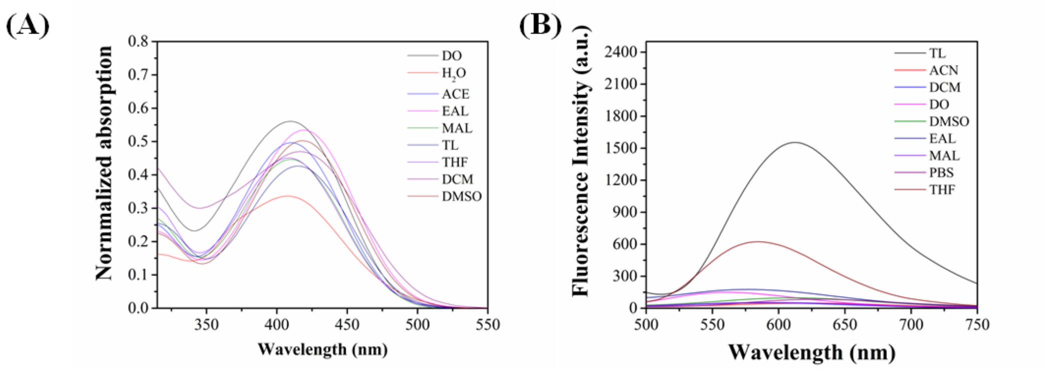

Figure S10. The absorption and emission spectra of **T-2** in 1,4-Dioxane, ethanol, water, acetone, methanol, toluene, tetrahydrofuran, dichloromethane and dimethyl sulfoxide.

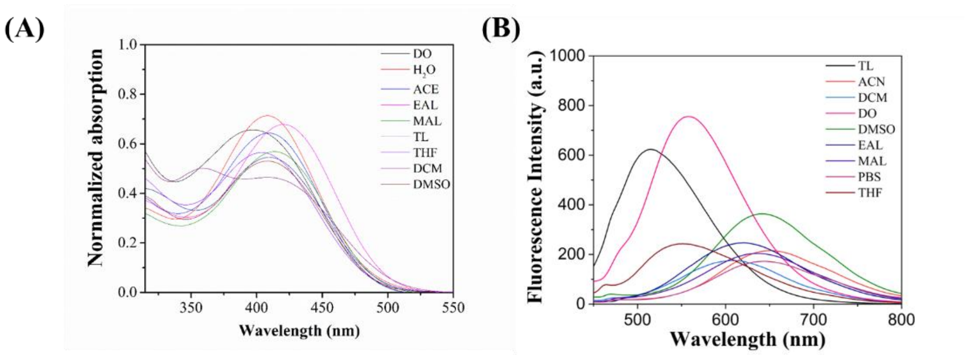

Figure S11. The absorption and emission spectra of **T-3** in 1,4-Dioxane, ethanol, water, acetone, methanol, toluene, tetrahydrofuran, dichloromethane and dimethyl sulfoxide.

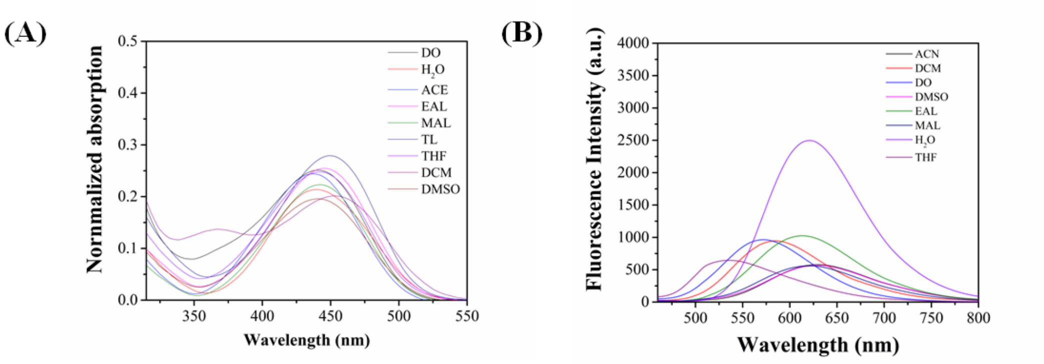

Figure S12. The absorption and emission spectra of **T-4** in 1,4-Dioxane, ethanol, water, acetone, methanol, toluene, tetrahydrofuran, dichloromethane and dimethyl sulfoxide.

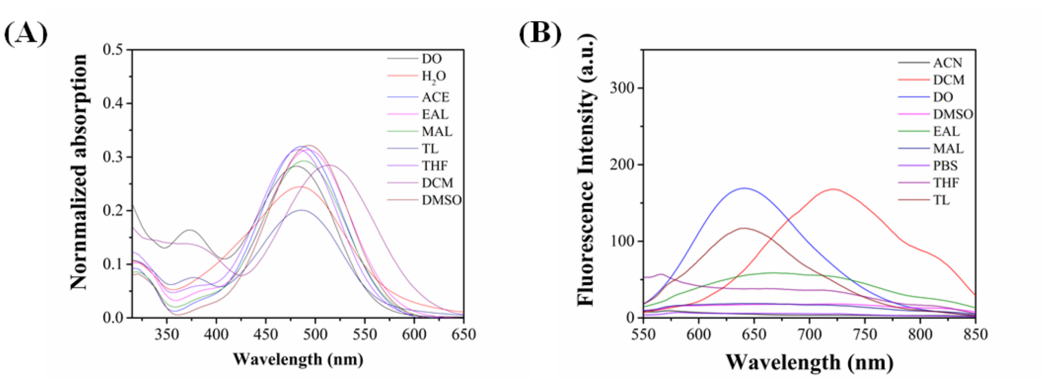

Figure S13. The absorption and emission spectra of **T-5** in 1,4-Dioxane, ethanol, water, acetone, methanol, toluene, tetrahydrofuran, dichloromethane and dimethyl sulfoxide.

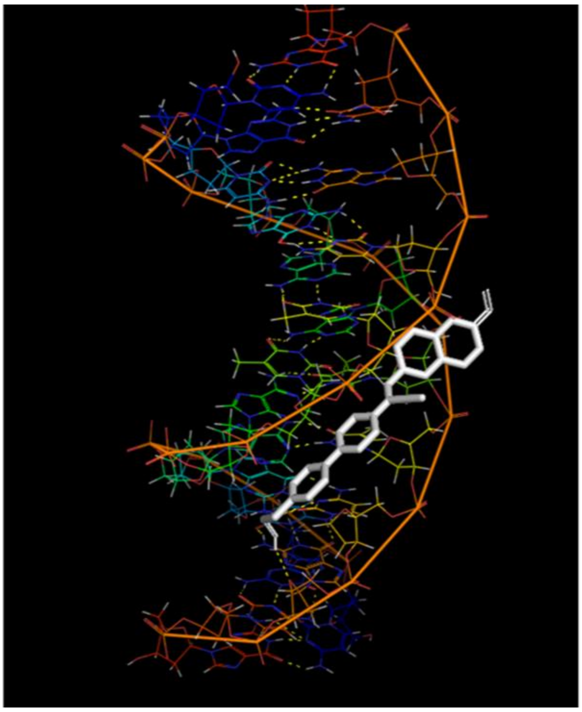

Figure S14. Calculated binding mode of **T-1** with RNA.

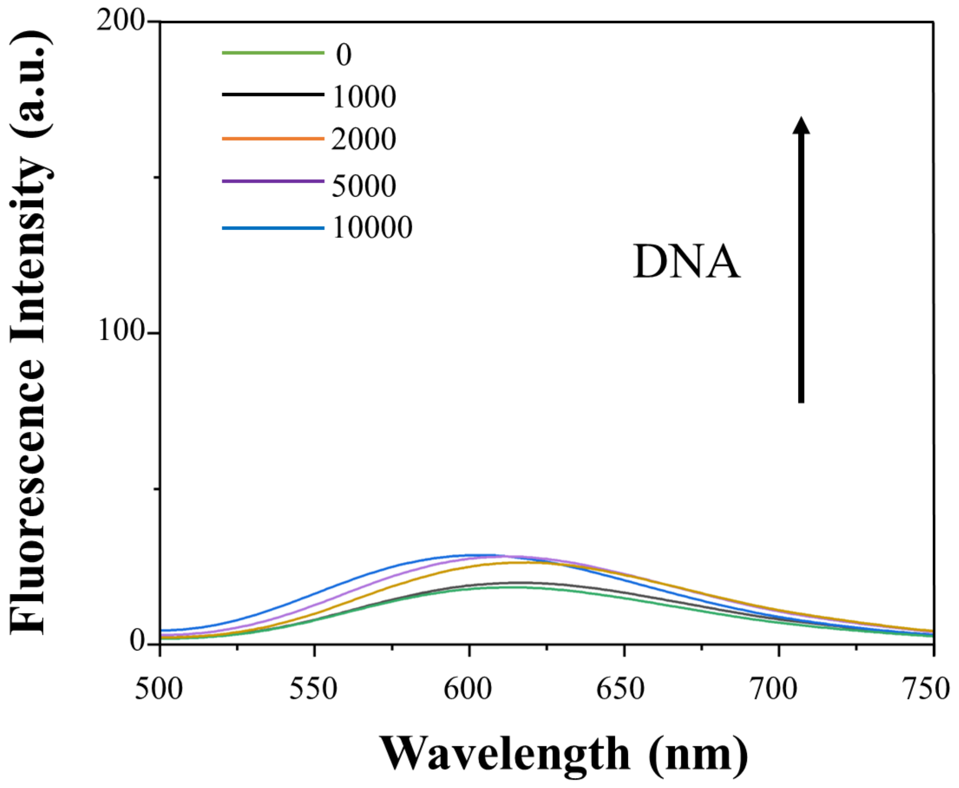

Figure S15. Fluorescence spectra of **T-1** (5μM) in the presence of 0−10000 equiv. DNA. λ_{ex} = 425 nm.

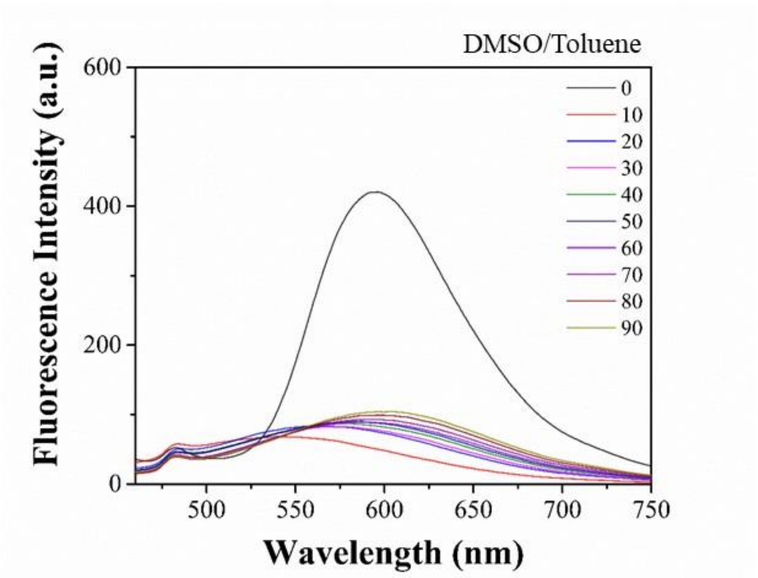

Figure S16. PL spectra of **T-4** in DMSO/Toluene mixtures with different DMSO fractions (λ_{ex}=450 nm).

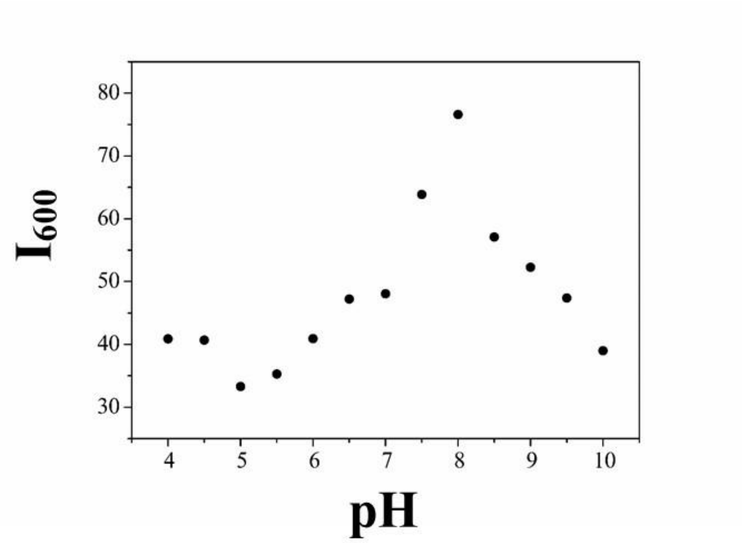

Figure S17. Emission spectra of **T-4** in PBS at different pH values.

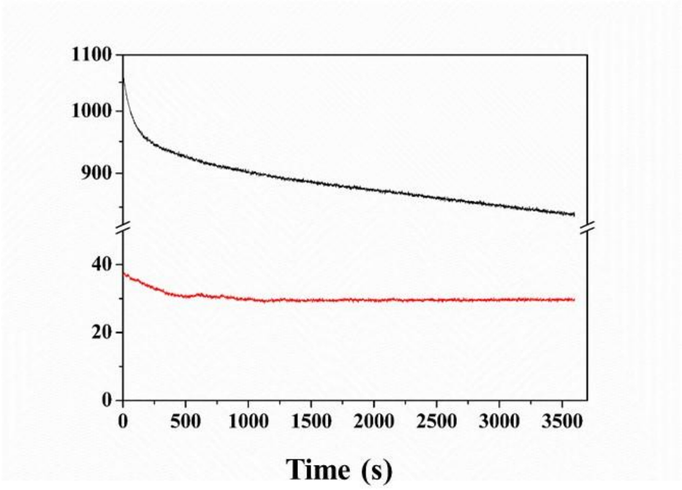

Figure S18. The photostability of T-4 and Indocyanine green in PBS.

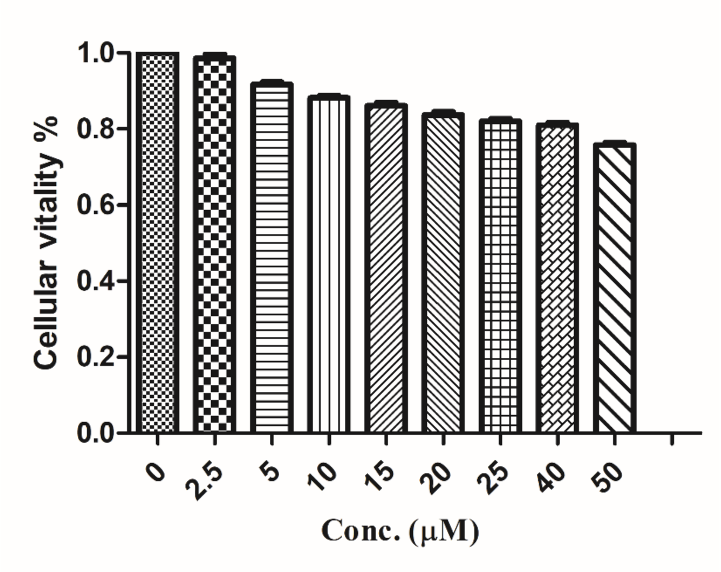

Figure S19. The cell viability of HeLa cells incubated with 1-50 μM of **DPA-SCP** for 24 h.

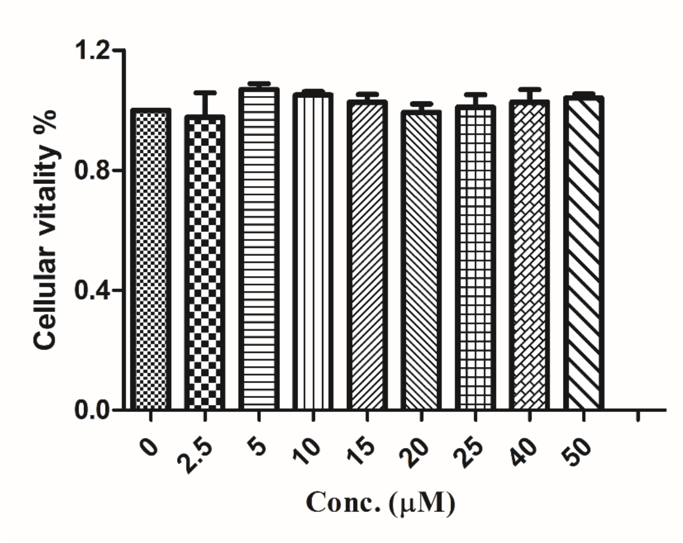

Figure S20. The cell viability of HeLa cells incubated with 2.5-50 μM of **T-4** for 24 h.

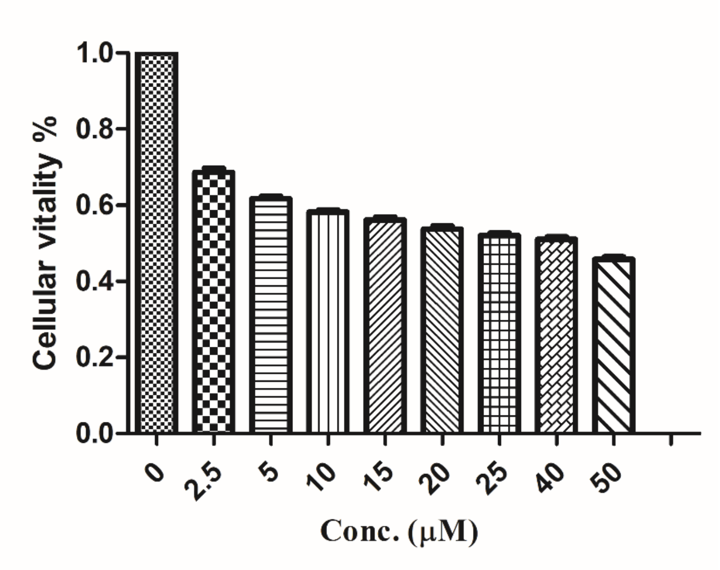

Figure S21. The phototoxicity of HeLa cells incubated with 2.5-50 μM of **DPA-SCP**.

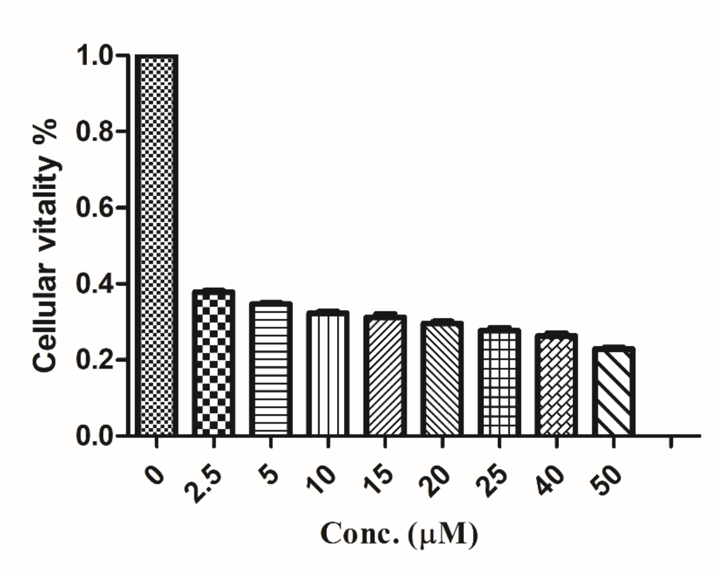

Figure S22. The phototoxicity of HeLa cells incubated with 2.5-50 μM of **T-4**.

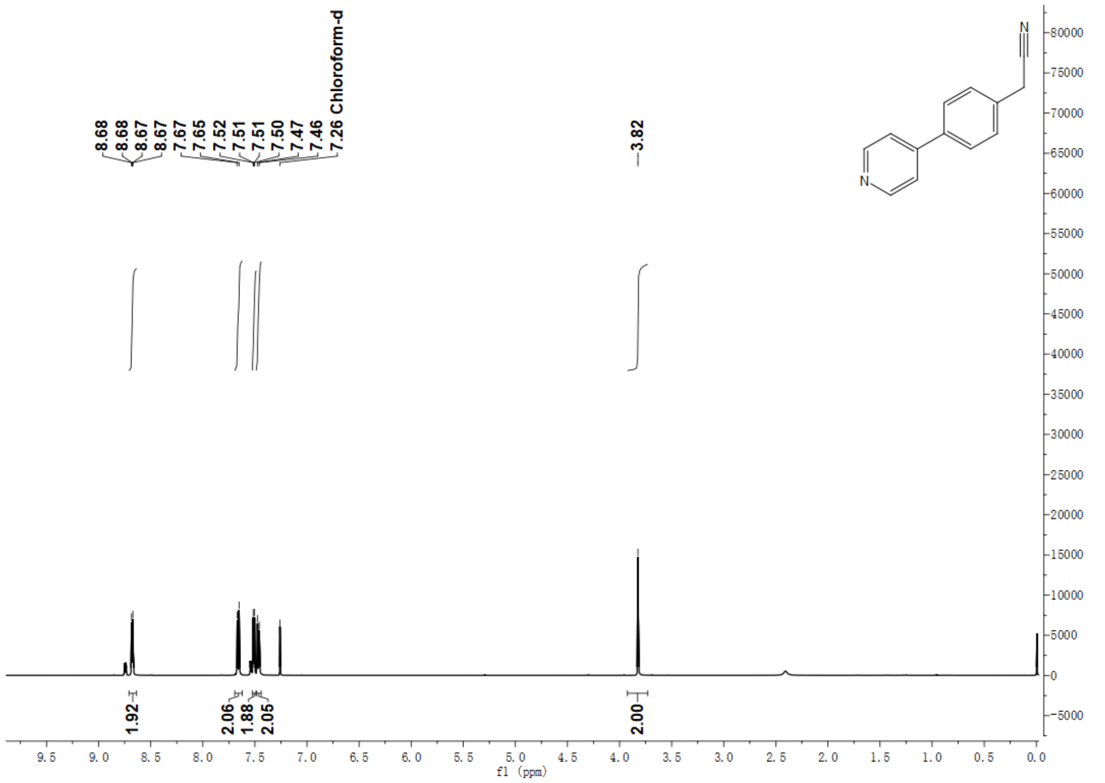

Figure S23. ^{1}H NMR spectrum of **P-1** in $CDCl_3$.

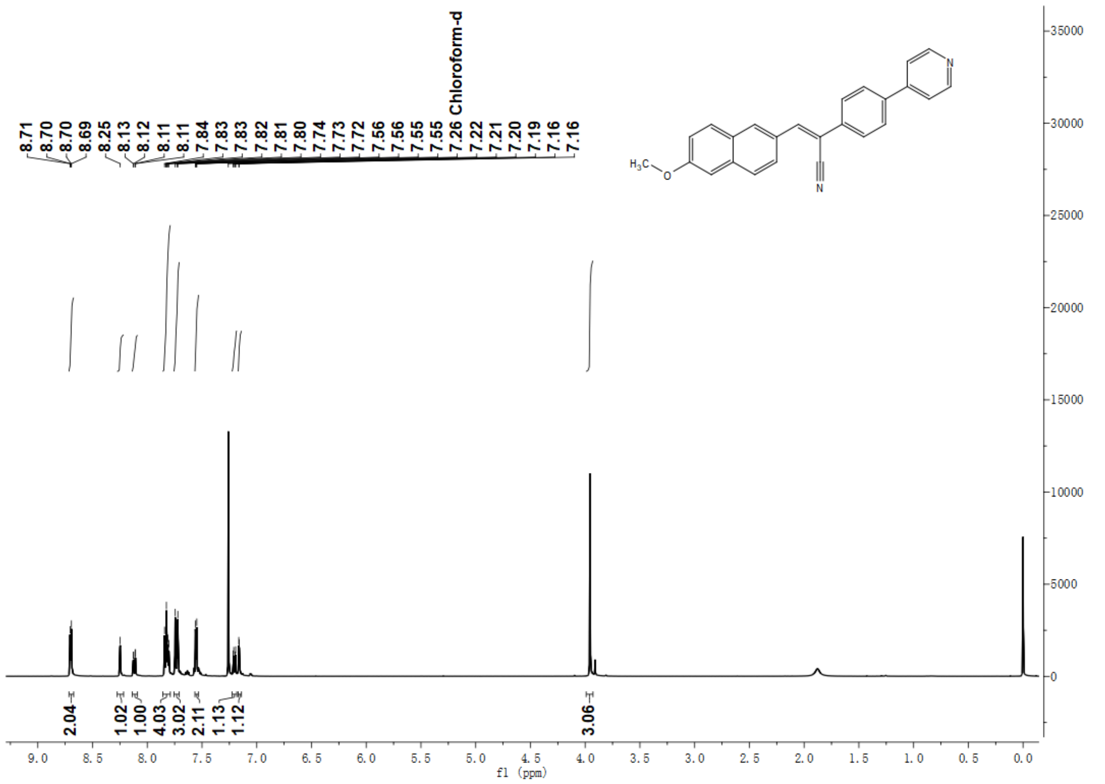

Figure. S24 ^{1}H NMR spectrum of **T-1-1** in $CDCl_3$.

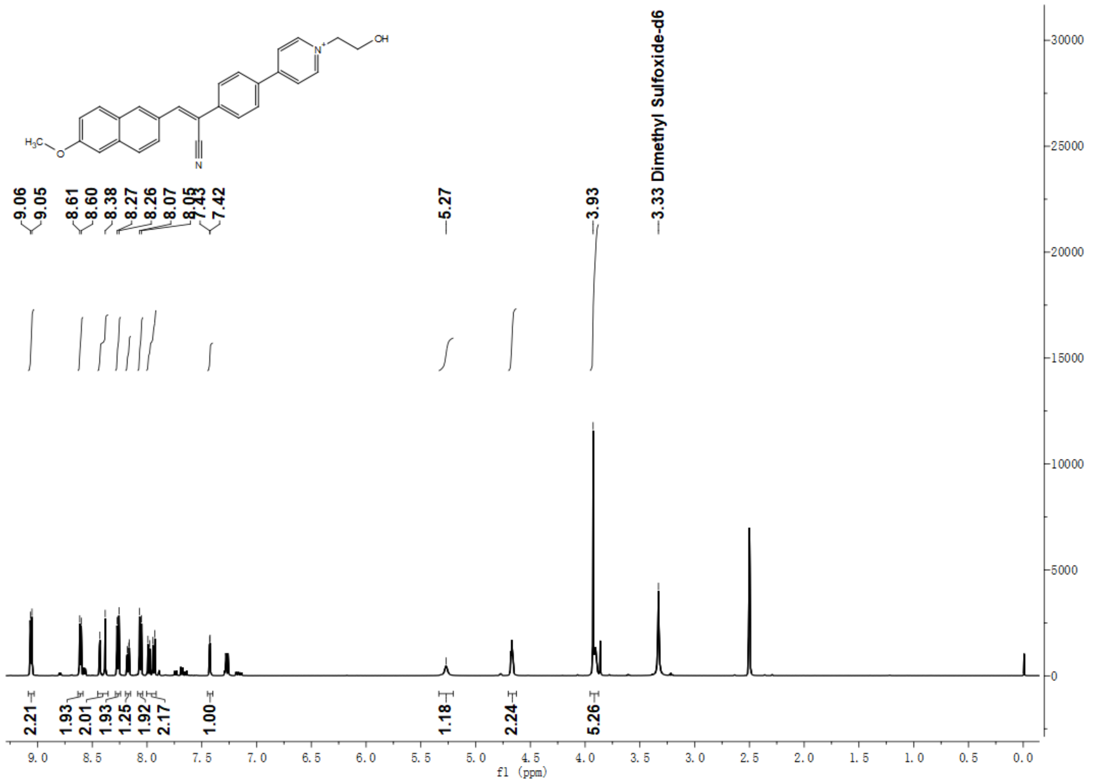

Figure. S25 ^{1}H NMR spectrum of **T-1** in DMSO-d_6.

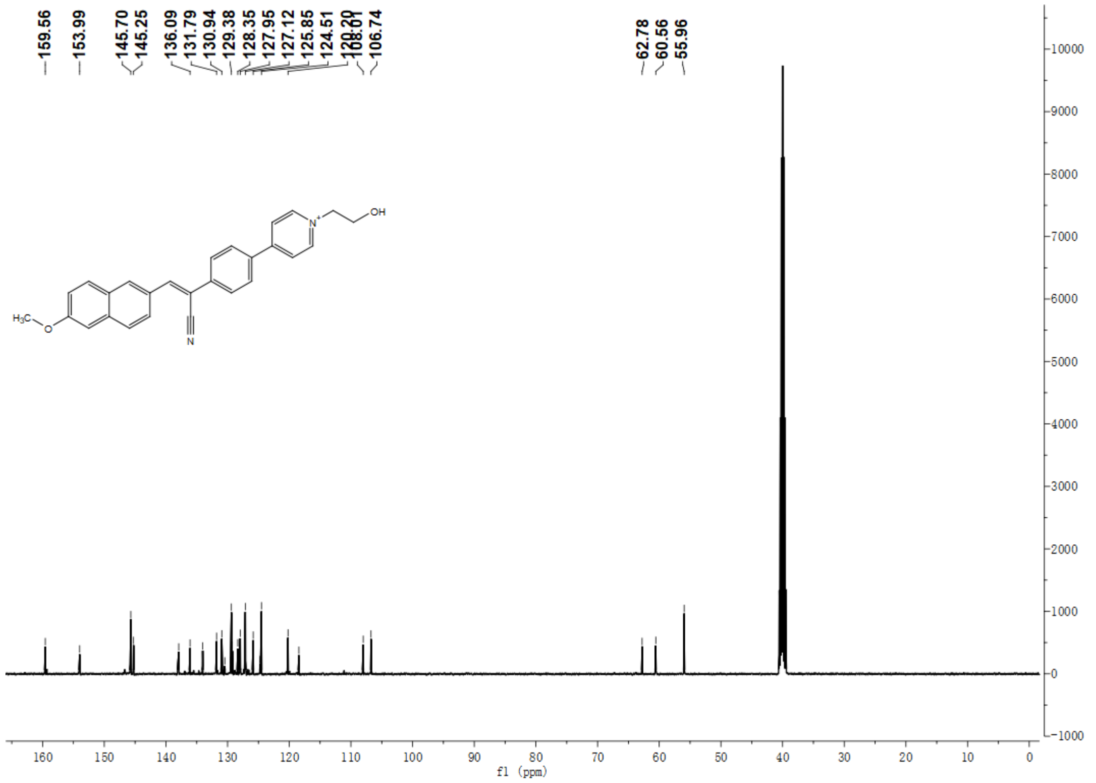

Figure. S26 The ^{13}C NMR spectra of **T-1** in DMSO-d_6.

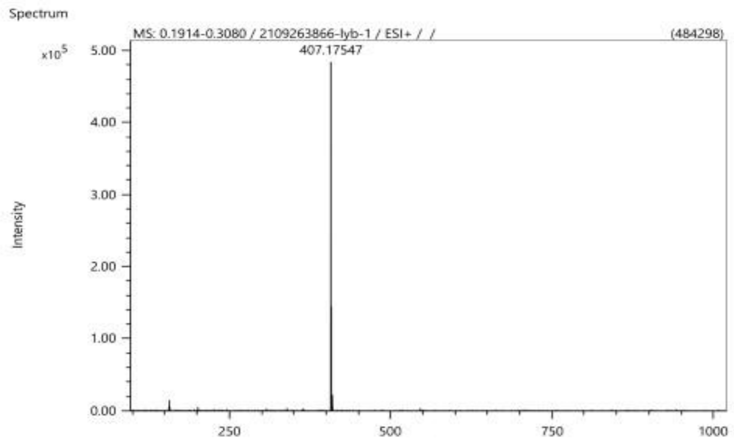

Figure. S27 The HRMS of **T-1.**

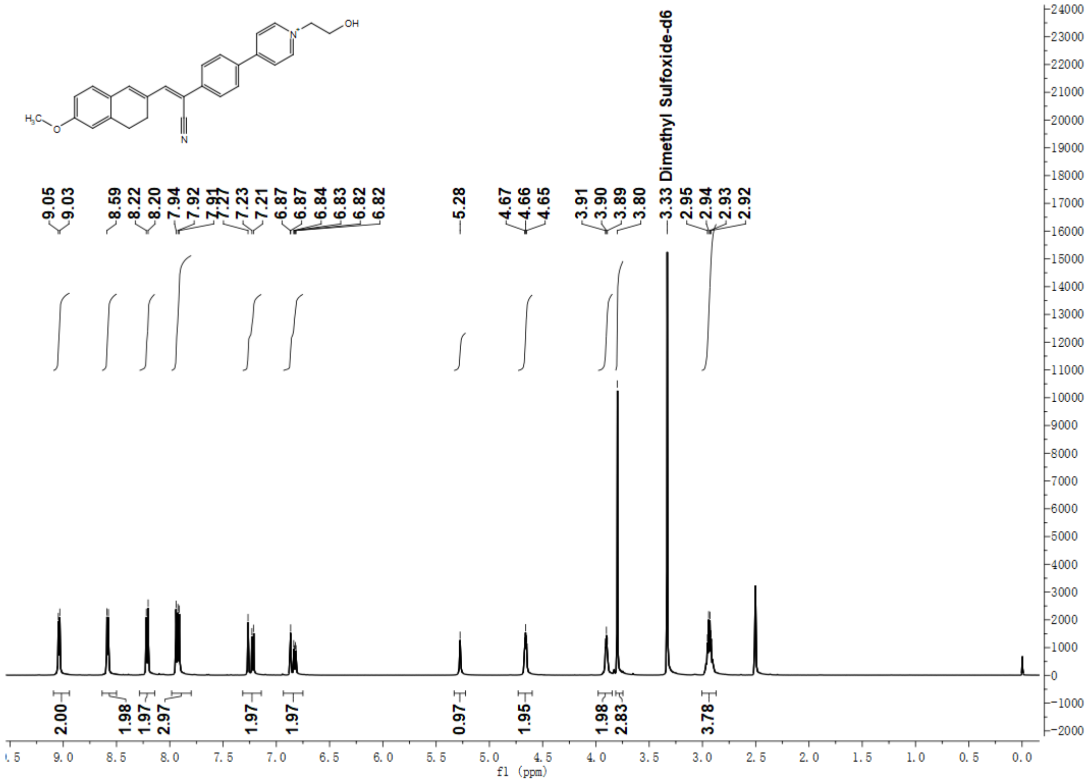

Figure. S28 ^{1}H NMR spectrum of **T-2** in DMSO-d_6.

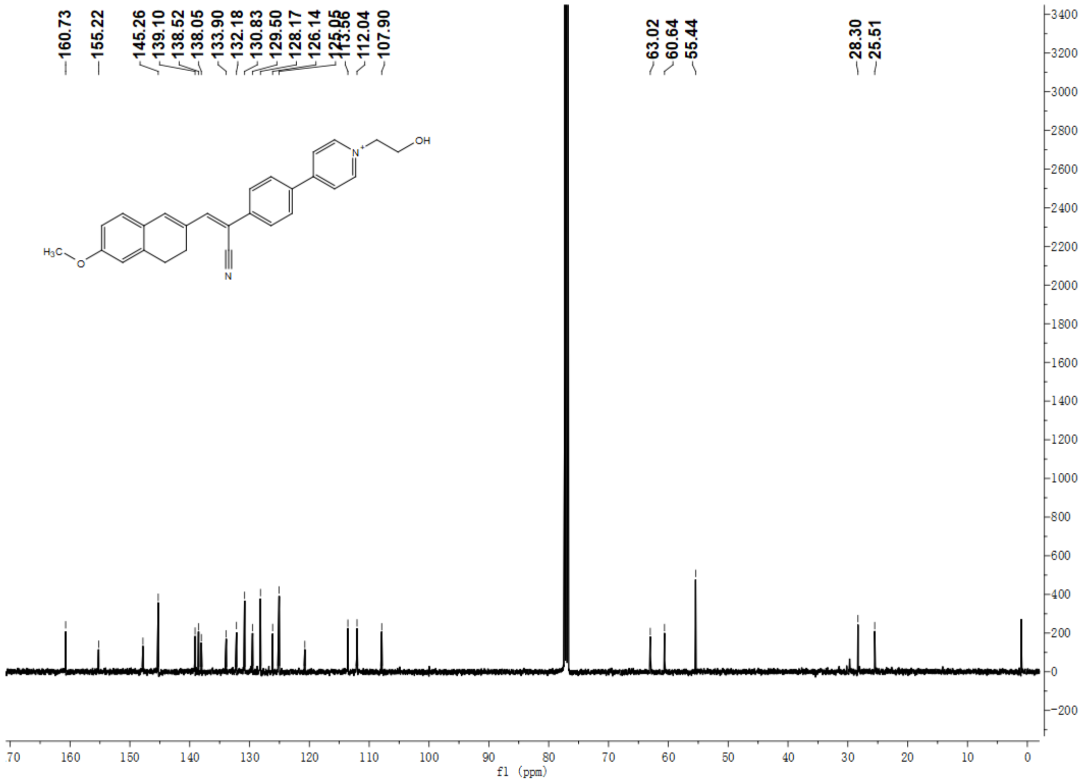

Figure. S29 The ^{13}C NMR spectra of **T-2** in DMSO-d_6.

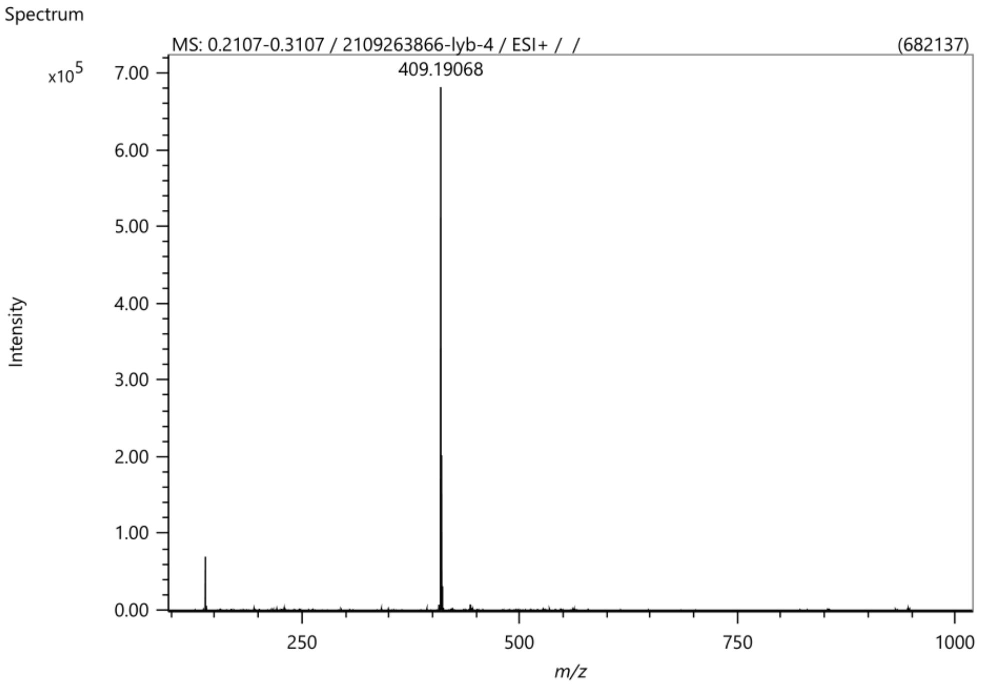

Figure. S30 The HRMS of **T-2.**

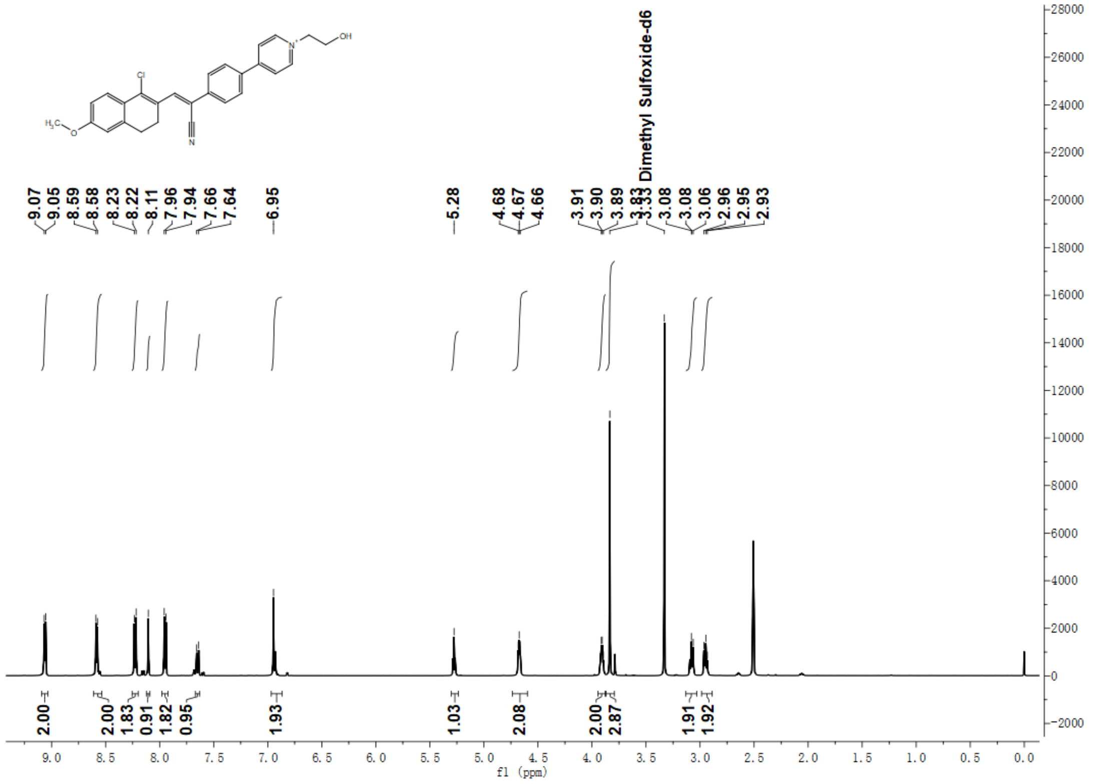

Figure. S31 ^{1}H NMR spectrum of **T-3** in DMSO-d_6.

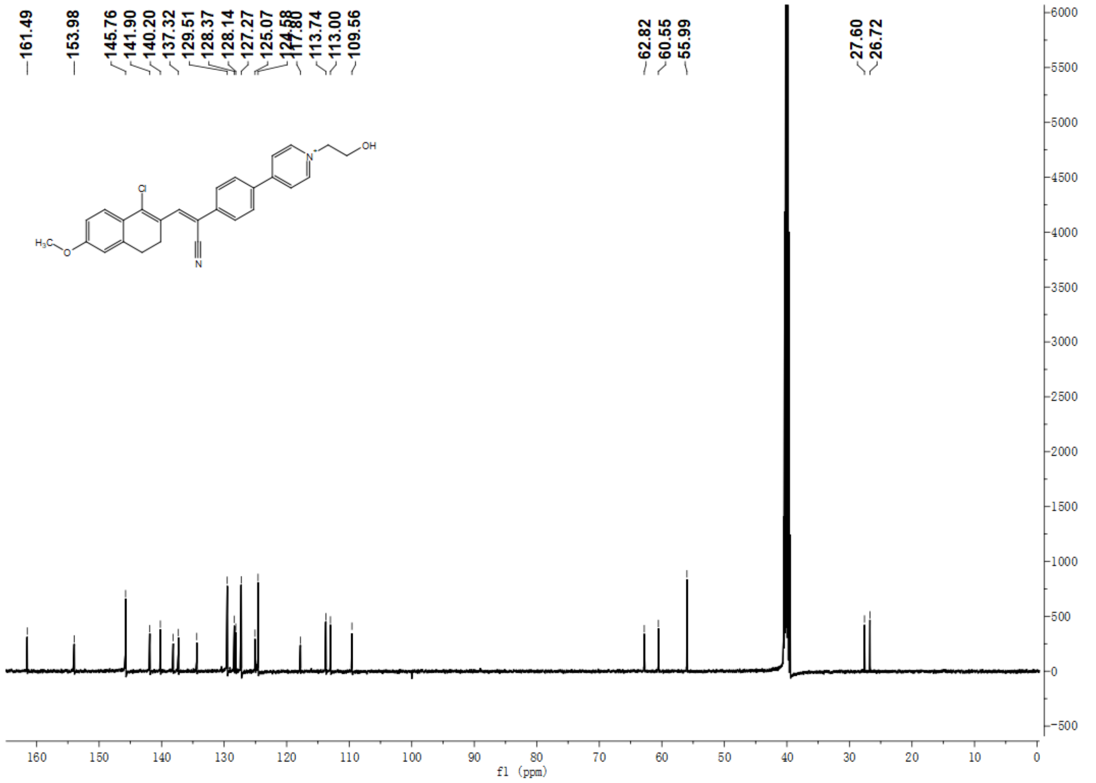

Figure. S32 The ^{13}C NMR spectra of **T-3** in DMSO-d_6.

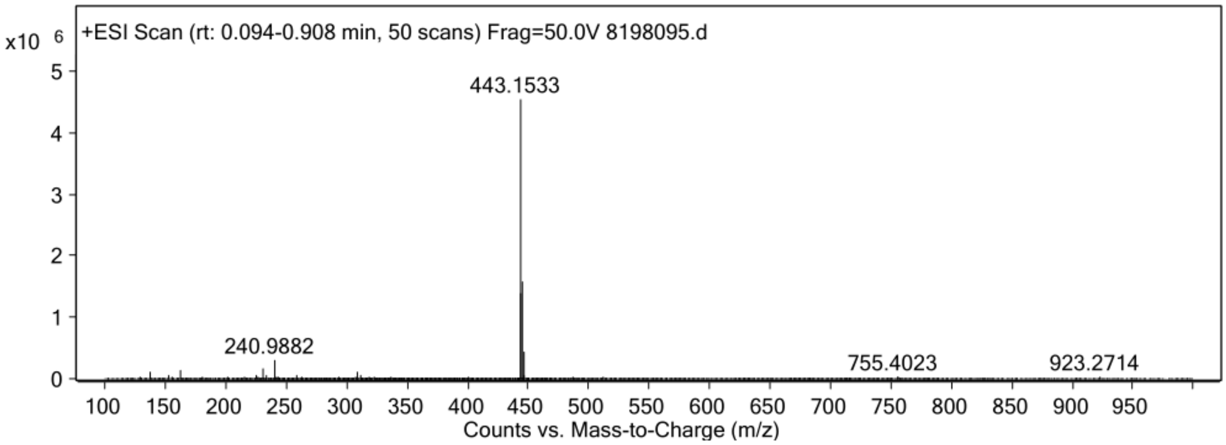

Figure. S33 The HRMS of **T-3.**

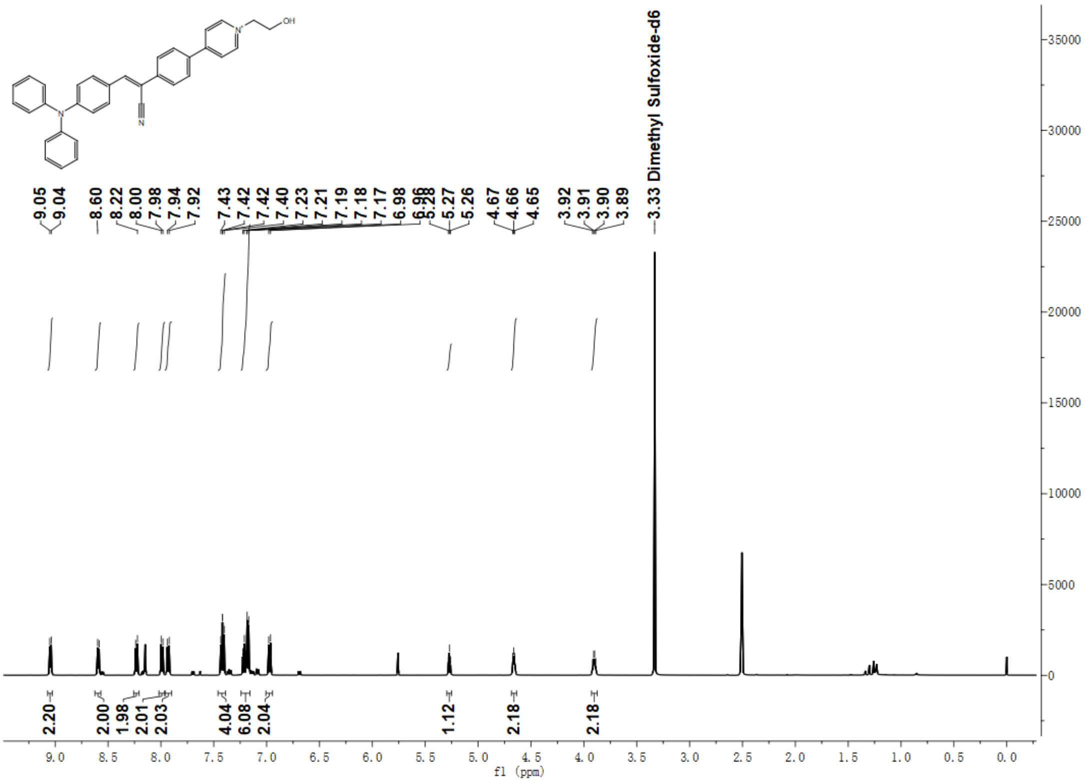

Figure. S34 ^{1}H NMR spectrum of **T-4** in DMSO-d_6.

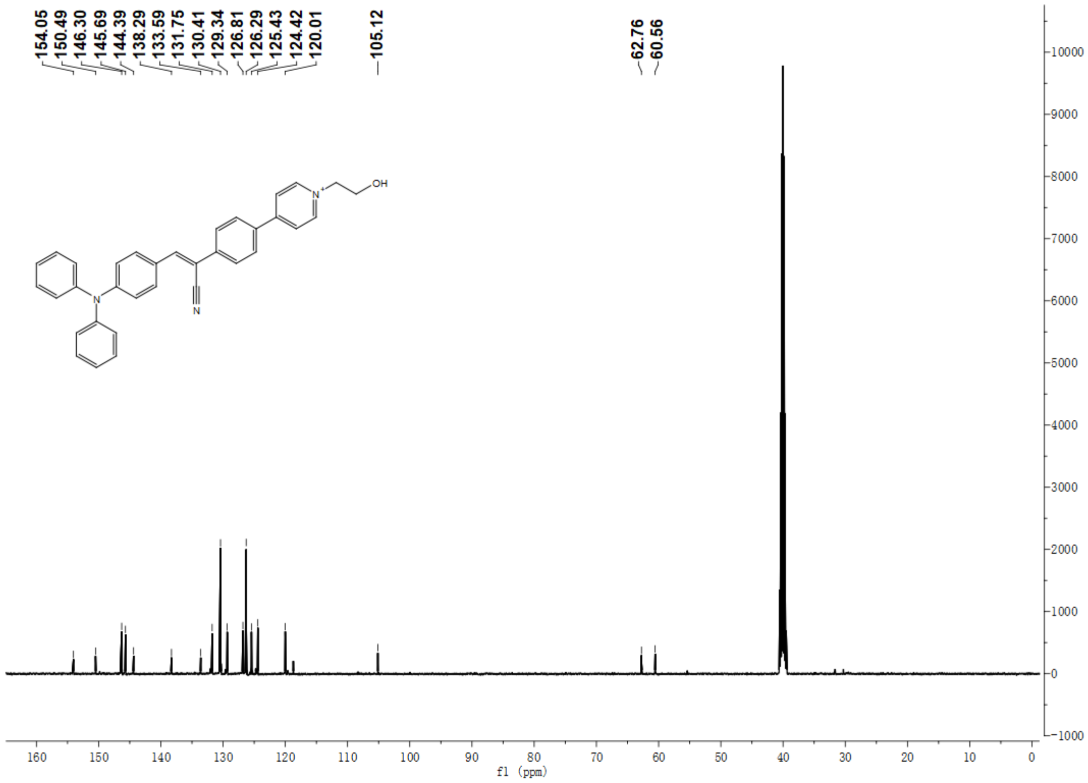

Figure. S35 The ^{13}C NMR spectra of **T-4** in DMSO-d_6.

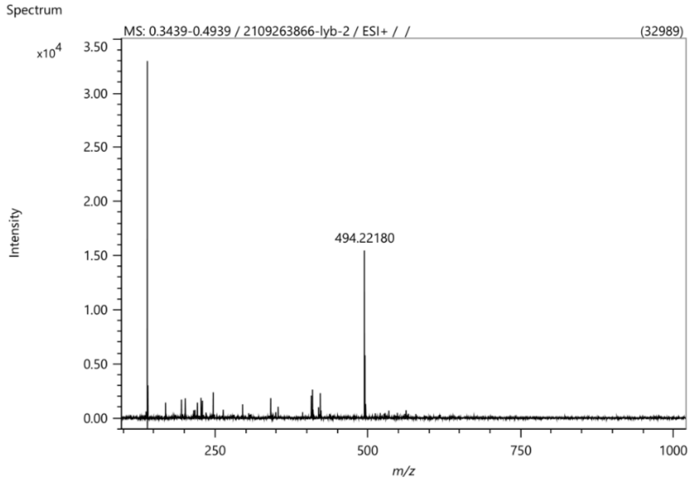

Figure. S36 The HRMS of **T-4.**

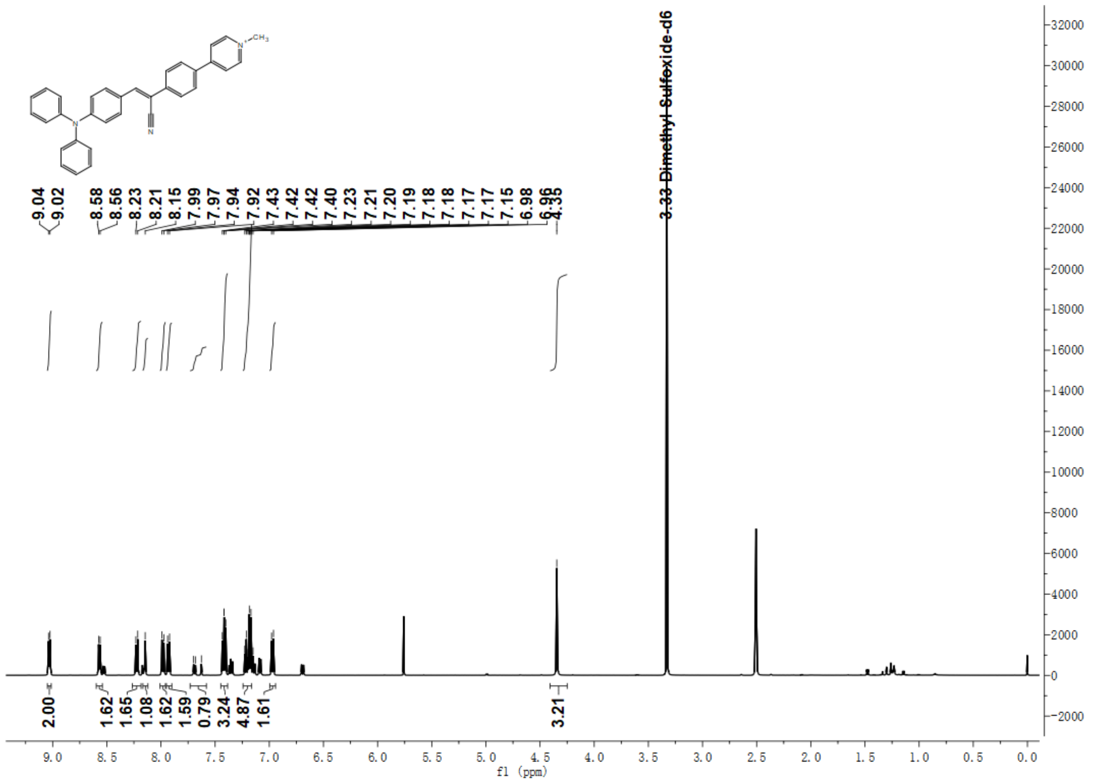

Figure. S37 ^{1}H NMR spectrum of **Mito-2** in DMSO-d_6.

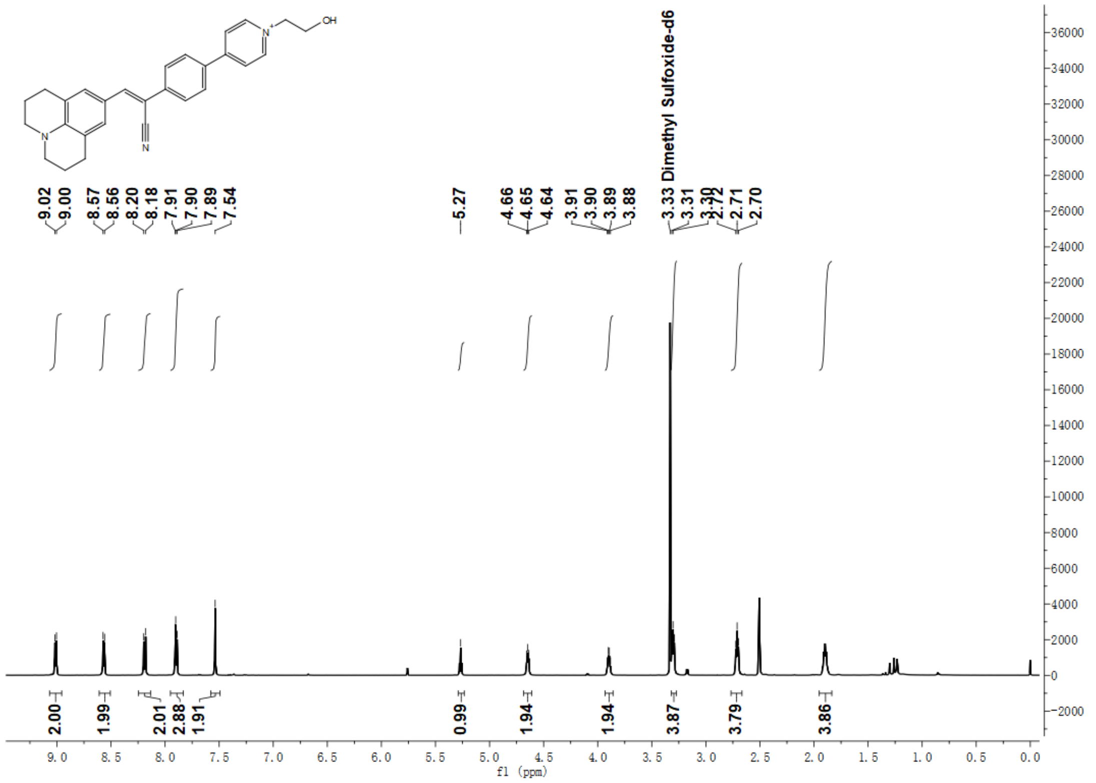

Figure. S38 ^{1}H NMR spectrum of **T-5** in DMSO-d_6.

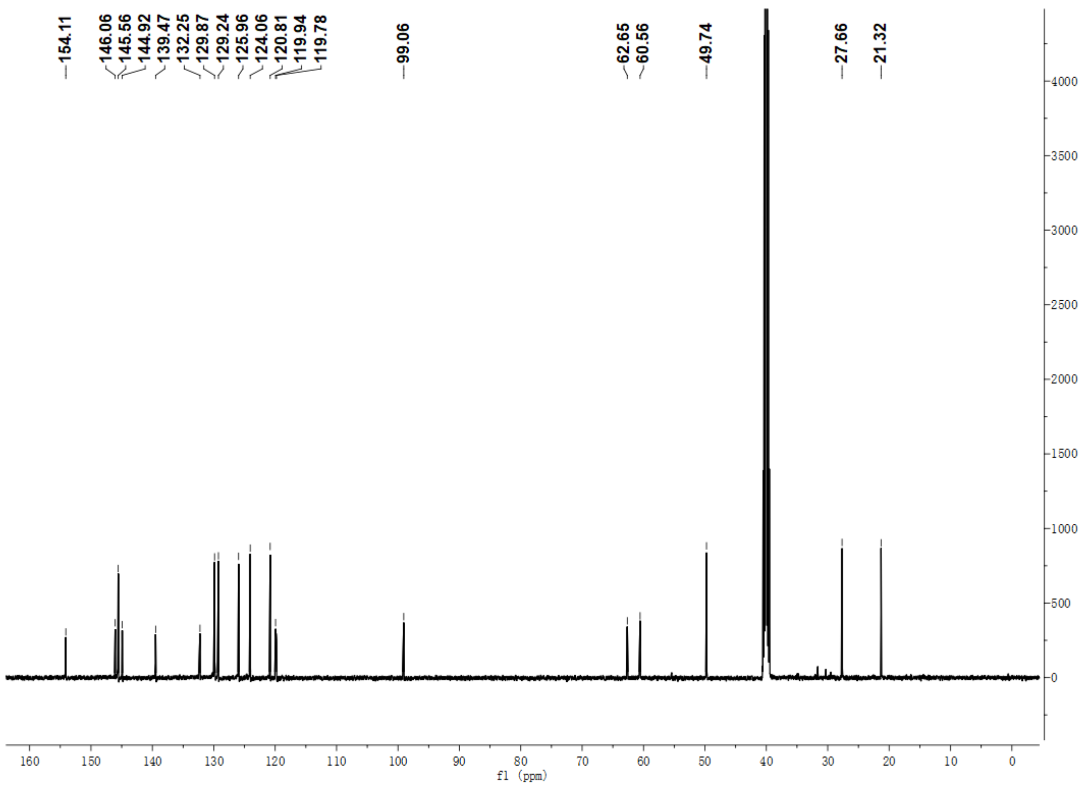

Figure. S39 The ^{13}C NMR spectra of **T-5** in DMSO-d_6.

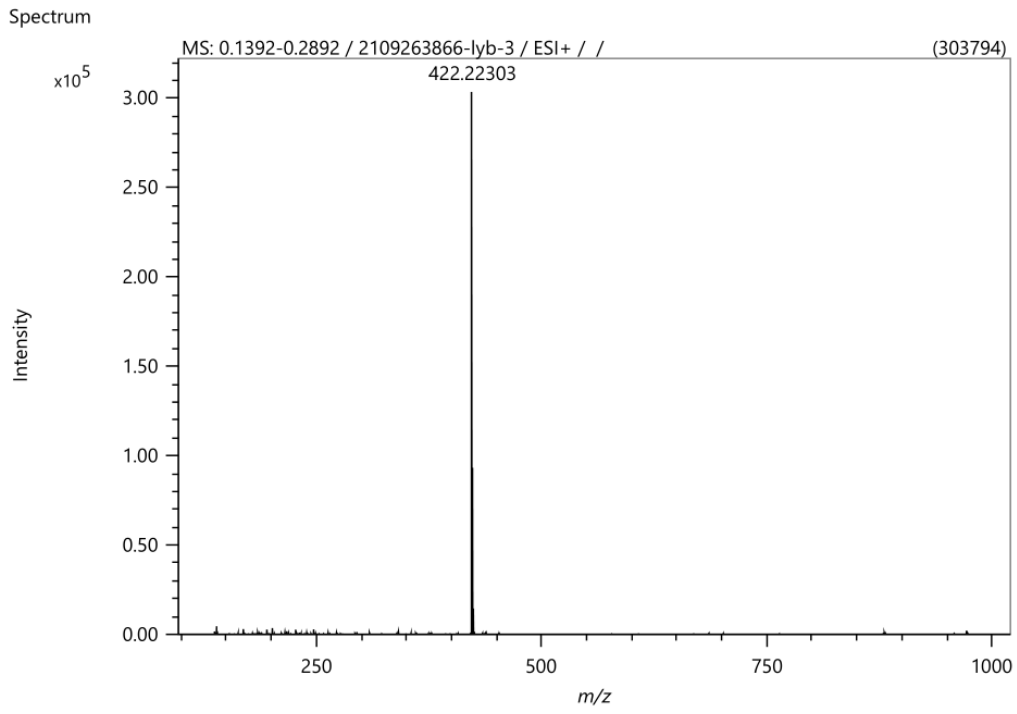

Figure. S40 The HRMS of **T-5.**

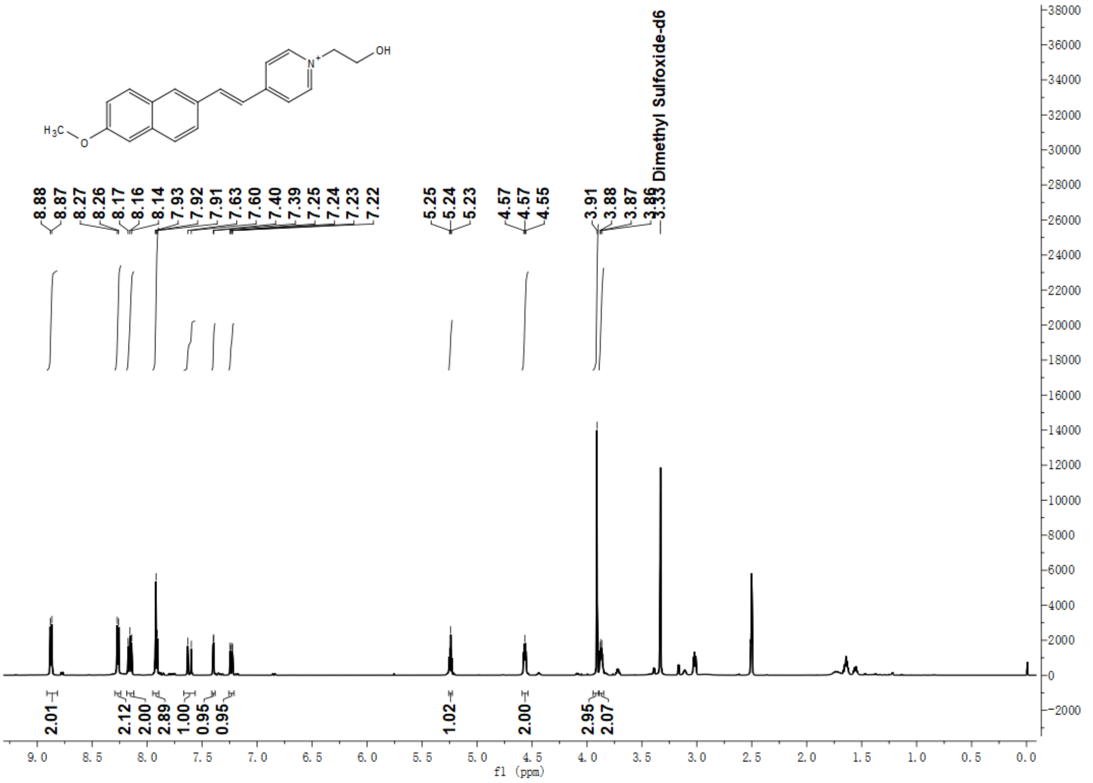

Figure. S41 ^{1}H NMR spectrum of **S-1** in DMSO-d_6.

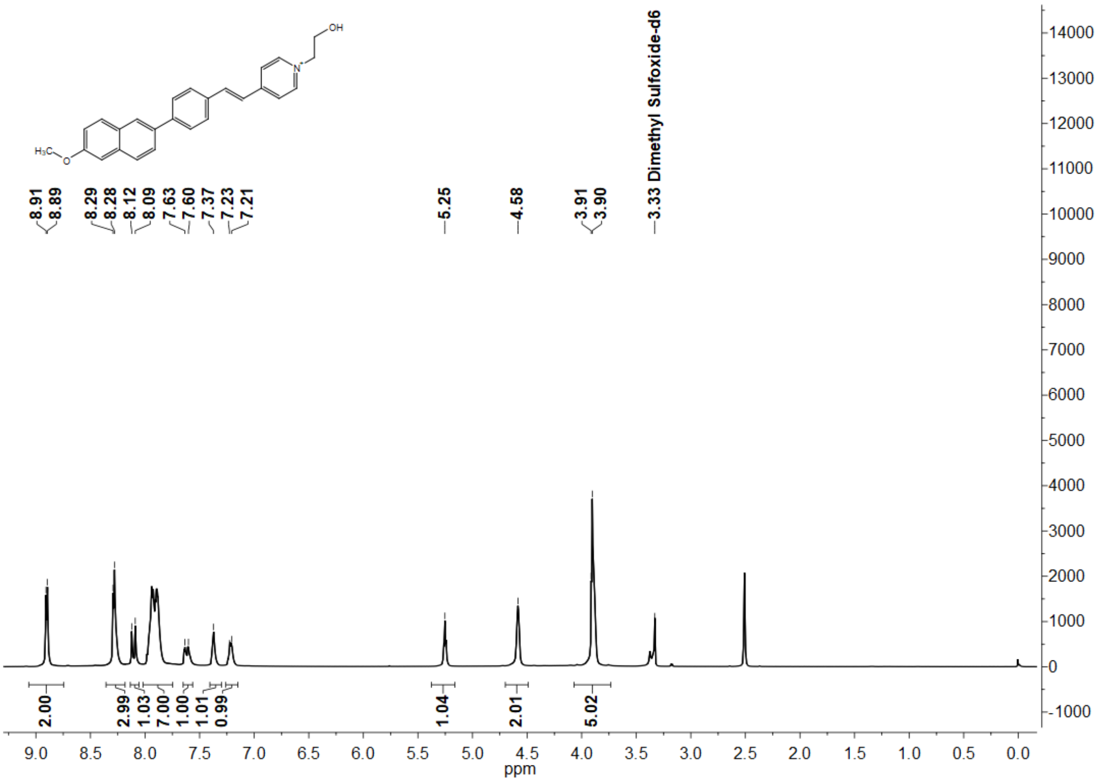

Figure. S42 ^{1}H NMR spectrum of **S-2** in DMSO-d_6.

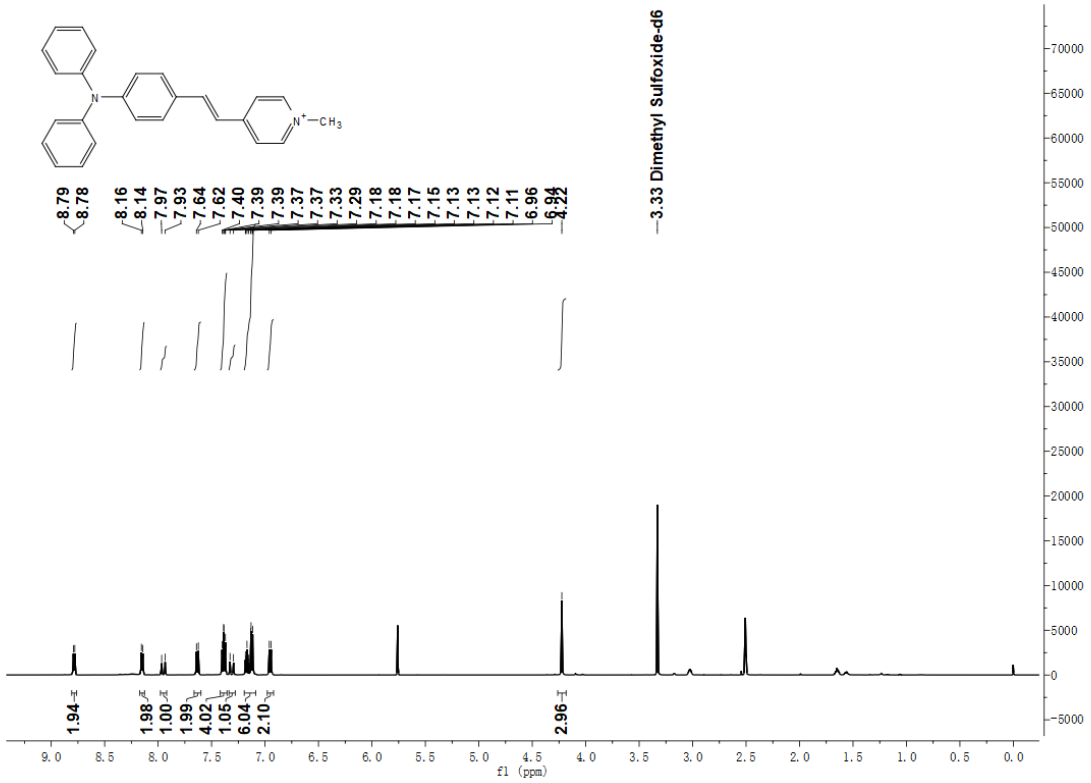

Figure. S43 ^{1}H NMR spectrum of **Mito-1** in DMSO-d_6.

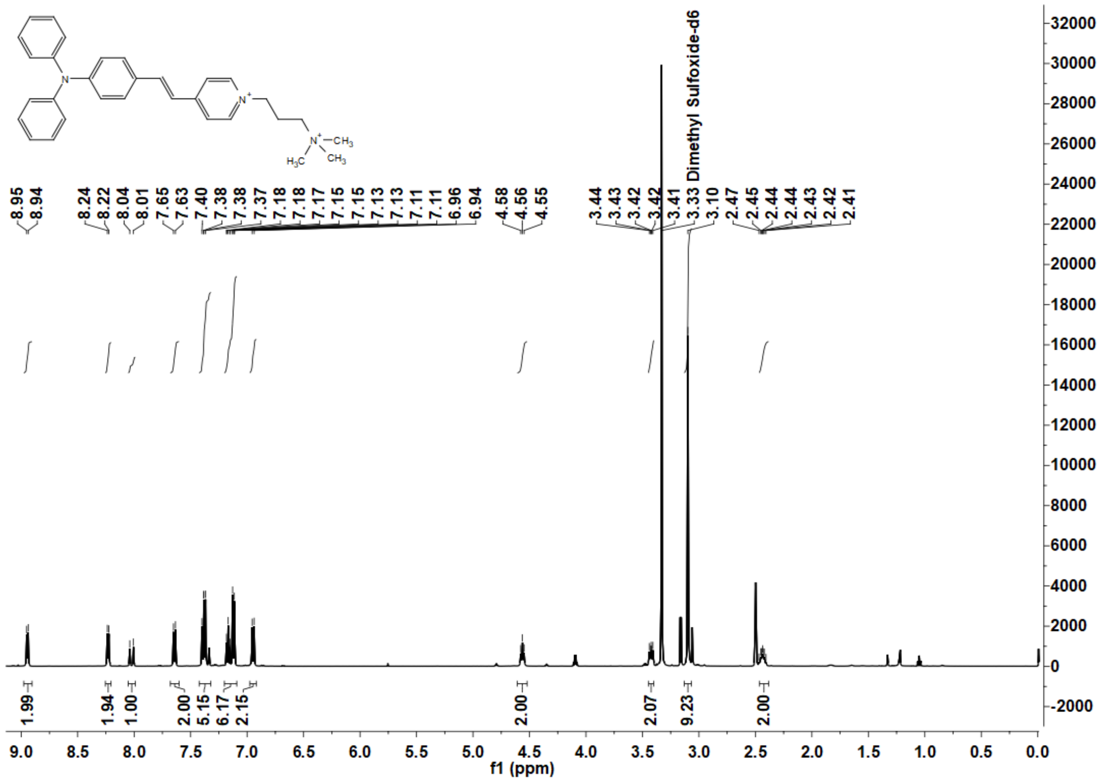

Figure. S44 ^{1}H NMR spectrum of **CM-1** in DMSO-d_6.

References

1.Cao, H., Yue, Z., Gao, H., Chen, C., Cui, K., Zhang, K., Cheng, Y., Shao, G., Kong, D., and Li, Z. (2019). In vivo real-time imaging of extracellular vesicles in liver regeneration via aggregation-induced emission luminogens. ACS nano 13, 3522-3533.

2.Han, X., Ma, Y., Chen, Y., Wang, X., and Wang, Z. (2020). Enhancement of the aggregation-induced emission by hydrogen bond for visualizing hypochlorous acid in an inflammation model and a hepatocellular carcinoma model. Anal. Chem. 92, 2830-2838.
